# Recruiting Mechanism and Functional Role of a Third Metal Ion in the Enzymatic Activity of 5′ Structure-Specific Nucleases

**DOI:** 10.1021/jacs.9b10656

**Published:** 2020-01-15

**Authors:** Elisa Donati, Vito Genna, Marco De Vivo

**Affiliations:** Laboratory of Molecular Modelling & Drug Discovery, Istituto Italiano di Tecnologia, Via Morego 30, 16163 Genoa, Italy

## Abstract

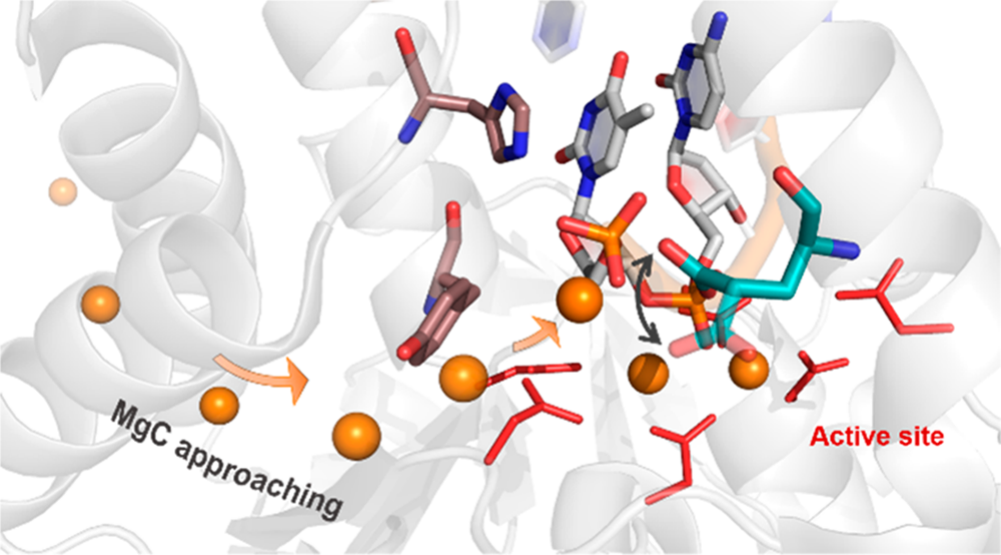

Enzymes of the 5′ structure-specific nuclease family are crucial for DNA repair, replication, and recombination. One such enzyme is the human exonuclease 1 (hExo1) metalloenzyme, which cleaves DNA strands, acting primarily as a processive 5′-3′ exonuclease and secondarily as a 5′-flap endonuclease. Recently, in crystallo reaction intermediates have elucidated how hExo1 exerts hydrolysis of DNA phosphodiester bonds. These hExo1 structures show a third metal ion intermittently bound close to the two-metal-ion active site, to which recessed ends or 5′-flap substrates bind. Evidence of this third ion has been observed in several nucleic-acid-processing metalloenzymes. However, there is still debate over what triggers the (un)binding of this transient third ion during catalysis and whether this ion has a catalytic function. Using extended molecular dynamics and enhanced sampling free-energy simulations, we observed that the carboxyl side chain of Glu89 (located along the arch motif in hExo1) flips frequently from the reactant state to the product state. The conformational flipping of Glu89 allows one metal ion to be recruited from the bulk and promptly positioned near the catalytic center. This is in line with the structural evidence. Additionally, our simulations show that the third metal ion assists the departure, through the mobile arch, of the nucleotide monophosphate product from the catalytic site. Structural comparisons of nuclease enzymes suggest that this Glu(Asp)-mediated mechanism for third ion recruitment and nucleic acid hydrolysis may be shared by other 5′ structure-specific nucleases.

## Introduction

Recent structural data have shown the recurring presence of a third metal ion close to the two-metal-ion center of nucleic-acid-processing enzymes.^1−4^ This third ion has been captured during different stages of catalysis of vital enzymatic reactions involved in DNA repair, recombination, and replication processes.^5−9^ These reactions are often related to cancer progression.^10−13^ Indeed, over the past few years, a third ion has been observed, or hypothesized, close to the two-metal-ion catalytic site of polymerases,^4^ nucleases,^14−18^ and topoisomerases.^19−21^ This suggests that the third metal ion may be actively involved in catalysis.^1,4,22^ However, there is debate over how this ion is recruited from the bulk and transiently binds the enzyme and how it could play a role in catalysis.

In this context, recent time-resolved in crystallo reaction intermediates^23^ have elucidated how human exonuclease 1 (hExo1) exerts its catalytic function, with sequential structures showing how the enzyme/DNA complex evolves during catalysis. hExo1 is an essential hydrolytic enzyme for genome maintenance. Belonging to the RAD2/XPG family,^24−30^ hExo1 is a 5′ structure-specific metallonuclease, which carries out a primary exonucleolytic activity on the 5′ recessed end and a secondary endonucleolytic cleavage on 5′-flap of the substrate DNA strand.^31,32^

**Figure 1:**
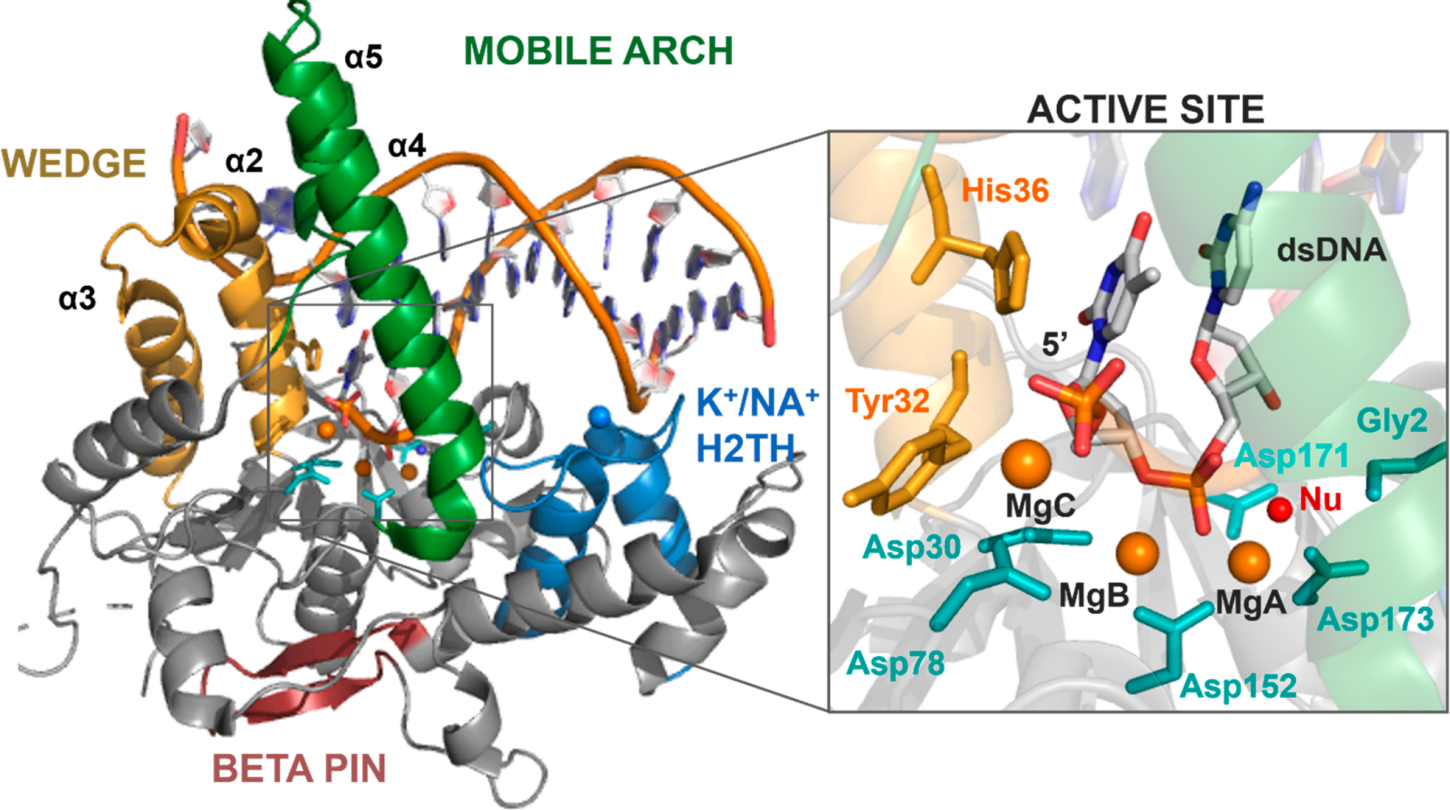
Catalytic domain of hExo1 in complex with DNA substrate and the two catalytic metal ions (PDB ID 5V06). (Left) hExo1 in cartoon and with colors for different structural motifs. (Right) Closer view of the active site. Three metal ions (MgA, MgB, MgC) are in orange, nucleophilic water molecule (Nu) is in red, two guide residues (Tyr32, His36) in yellow, and residues of the catalytic pocket (Gly2, Asp30, Asp78, Asp152, Asp171, Asp173) are in cyan. Scissile phosphate is correctly positioned for the nucleophilic attack, and MgC is coordinated by the 5′ terminal phosphate.

The structures show the enzymatic mechanism for DNA hydrolysis in hExo1, which starts in the precatalytic state with the intact double-strand DNA (dsDNA) recognized and bound (tethered) to the helix-two-turn-helix (H2TH) motif and to a monovalent (K^+^/Na^+^) ion state in hExo1 (Figure 1). Then catalysis begins with formation of the assembled active site, where the dsDNA bifurcates into the 5′ and 3′ single strands (i.e., the dsDNA “junction”). At this point, the scissile phosphate of the processed single 5′ strand is properly located at the reactive metal center at the N-terminal domain. In this state, the catalytic residues Lys85 and Arg92 interact with the scissile phosphate,^33^ after a rotation (clamped conformation) of the *mobile* helical arch formed by two α-helices (α4-α5) located near the junction. Here, the side chain of the guide residues Tyr32 and His36 are also rotated (Figure 1). These structural motifs contribute to the “threading mechanism”, whereby the 5′-flap DNA passes through the helical arch.^34^ In this way, basic residues steer the phosphate of the 5′ strand, promoting the proper location for hydrolysis of the scissile phosphate on top of the two catalytic ions, as expected for the recognized two-metal-ion mechanism.^34−40^

At this point, hydrolysis of the 5′ recessed end or the 5′-flap DNA substrate occurs.^41−43^ In hExo1, this is proposed to be favored by a structured network of interactions involving Arg95, Arg96, Arg121, and Asn124, which are located along the mobile arch. In particular, a key role in phosphate steering is proposed for the Arg96 and Asn124, which interact with the phosphate next to the scissile one (i.e., the terminal 5′ phosphate in the 5′ recessed-end substrate). This is similar to what has been observed in the enzyme hFEN1.^34^ After DNA hydrolysis, the nucleotide monophosphate group can leave the active site, with the “free” enzyme that now has Tyr32 and His36 back in their initial conformation.

**Scheme 1:**
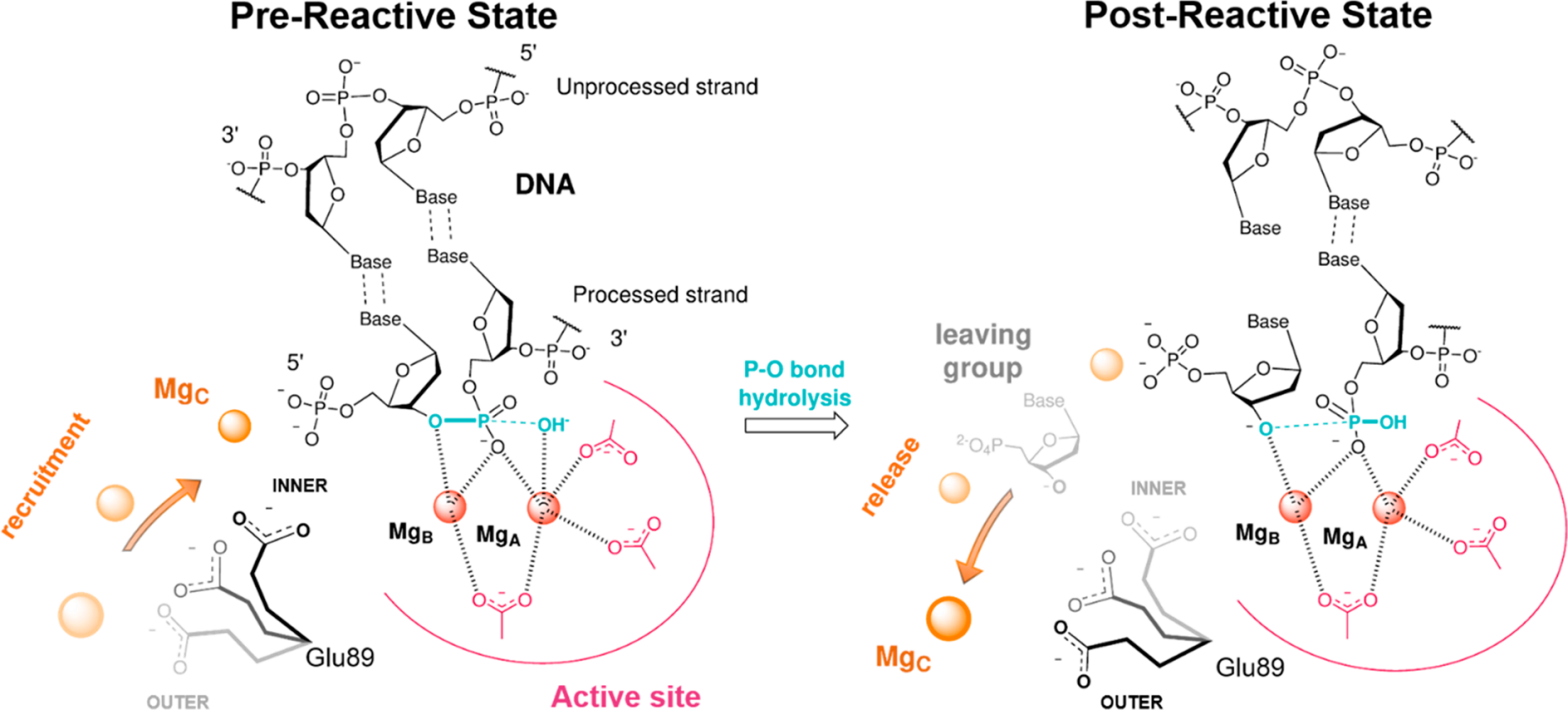
Schematic Representation of the Motion of the Transient Third Metal Ion, Which We Found to Be Intermittently Recruited/Released by Glu89 (switching its inner/outer conformations) during Exonuclease Catalysis This structural evidence suggests that the transient third ion may be crucial for substrate hydrolysis and/or leaving group departure.

Remarkably, these structural data show a transient third metal ion that is intermittently located close to the catalytic site during exonuclease catalysis (Scheme 1). This suggests that the transient third ion may play a role in substrate hydrolysis and/or leaving group departure.^44^ Indeed, during hExo1 catalysis, four different structures of the assembled active site were solved in the presence of a second-shell and solvent-exposed third metal ion, preserved close to the two-metal ion center (Figure S1). This ion is not found in the structure of the cleaved product, demonstrating its transient nature during catalysis.^1,23^

Here, we used force-field-based molecular dynamic (MD) simulations coupled to enhanced sampling free-energy calculations to investigate the role of the third ion during hExo1 catalysis. We compared several systems of wild-type and mutated hExo1 from the reactant state to the product state. We found that the second-shell and conserved Glu89 residue selects, recruits, and places the third ion close to the two-metal-ion catalytic site. We show that this negatively charged residue is functional and conserved among Exo1 belonging to different organisms and that this enzymatic mechanism is likely shared by other nucleases.

## Results

### Glu89 Selects, Recruits, and Places a Third Ion Close to the Catalytic Site of hExo1

First, we ran multiple unbiased force-field-based molecular dynamic (MD) simulations (∼700 ns in total) of the wild-type (*wt*) reactant state (Figure S2). We considered the enzyme in the reactive state, replacing the nonreactive Mn ions in the prereactive crystal structure (PDB ID 5V06)^23^ with native Mg ions.^31^ Notably, this structure features a third Mg ion (MgC) close to the two-metal-ion catalytic site. This ion is bound to the 5′ phosphate of the processed strand.

Residues Tyr32 and His36 are thought to guide substrate binding through interactions with their flexible side chain.^23^ Located above the reaction center, these residues maintain the crystallographic conformation, in which their side chain points “down” toward the two catalytic ions MgA and MgB beneath it (Figure S3). These ions maintain an octahedral coordination^45^ throughout the simulated time scale (i.e., 360 ns). As a result, the nucleophilic water molecule remains optimally positioned for nucleophilic attack, sitting on top of MgA, in front of the substrate’s scissile phosphodiester bond, at 3.88 ± 0.11 Å (Figure S3).

Despite the overall stability of the protein–DNA complex, the side chains at the base of the mobile arch (i.e., at the gateway) sample different conformations. In particular, the initial clamped conformation shows some flexibility over time, with Lys85 moving slightly further away from the scissile phosphate (∼6 Å vs 4 Å in PDB ID 5V06 see Figure S4). Moreover, we observed an amplified mobility of MgC, reflected in its enhanced RMSD of 1.63 ± 0.42 Å, as compared to the two catalytic ions and its anchor 5′ nucleotide, which are highly stable with an RMSD of 0.54 ± 0.17 and 0.82 ± 0.18 Å, respectively. Indeed, MgC moves from its initial position, forming new interactions with the flexible Glu89 carboxylate along the α-helix of the gateway. As a result, the mobile MgC alternates between a bidentate and a monodentate binding coordination with the 5′ phosphate and Glu89 (Figure S5), always maintaining an octahedral shell. In this regard, the motions of the Glu89 side chain are described by the pseudodihedral angle ϕ (taken along the N, Cα, Cδ, Cγ bonds; see Figure S5), which oscillates from ∼120° to ∼−10° (Figure S5), never reaching a conformation of the product state (−36°, PDB ID 5V0A).^23^ Concomitantly, we observed that Arg96, which is located along the mobile arch, shortens its distance from the 5′ phosphate, reaching a value of ∼4.5 ± 0.19 Å (compared to an initial distance of 6 Å in PDB ID 5V06). This distance corresponds well to the value in the crystal structure of the product state (5.1 Å, PDB ID 5V0A) (Figure S6). Taken together, these results support the hypothesis of a gradual removal and possible departure of the third ion during catalysis, as suggested by comparing the structural data of the reactant (MgC present, PDB ID 5V06) and product states (MgC missing, PDB ID 5V0A).

To further characterize the structural impact of MgC bound close to the two-metal-ion catalytic site in the reactant state, we manually removed it and ran multiple simulations of the solvated system (∼1 μs, in total). This protein–DNA complex showed no major difference in the overall backbone stability (see Figure S2) compared to the three-ion reactant state (see above). However, in these replications, the catalytic residues Lys85 and Arg92 maintained their native H-bond pattern with the scissile phosphate (as in the crystallographic structure) for the whole simulation. Thus, the catalytic residue Lys85 behaved differently to the three-ion system. Also, in these simulations, at times Glu89 interacted transiently with Arg93, located along the mobile arch. This interaction was observed only when Glu89 adopted the outer conformations, in the absence of MgC. Concurrently, Arg96 maintained its starting orientation and never interacted with 5′ phosphate (Figure S6). Again, this differs from the observations in the presence of MgC (see previous paragraph).

**Figure 2:**
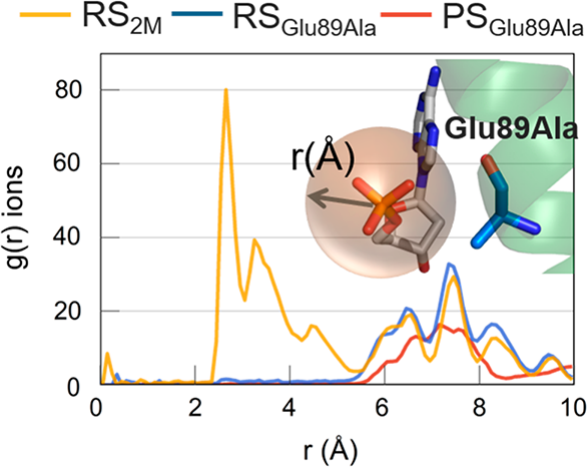
Radial distribution function, *g*(*r*), calculated for ions around 10 Å from the center of mass of the 5′ phosphate group. Plot shows the presence of ions ∼3 Å from the 5′ phosphate group for the RS_2M_ system. In this system, a K^+^ ion approached the negatively charged group. For the RS_Glu89Ala_ and PS_Glu89Ala_ systems, there are no ions within ∼5.5 Å of the 5′ phosphate, as indicated by the *g*(*r*) values of ∼0. In the upper right corner, the 5′ phosphate group and Glu89Ala residues are shown in licorice (taken from the PS_Glu89Ala_ simulations).

Intriguingly, during the initial equilibration phase (∼10 ns), the side chain of Glu89 undergoes a marked rearrangement from the initial inner conformation to an outer conformation toward the bulk water. This rotation is captured well by the Glu89 pseudodihedral angle ϕ, which changes from positive values of ∼+100° (inner) to negative values of ∼−95° (outer) (Figure S7). Importantly, in this new solvent-exposed conformation, we observed that Glu89 carboxylate transiently recruits and binds monovalent ions from the bulk (either K^+^ or Na^+^, freely diffusing in solution). This result is also confirmed by the radial distribution function, *g*(*r*), calculated as the variation of the density of the ions from the center of mass of the 5′ phosphate group (Figure 2), which displays two peaks at ∼2.7 and ∼3.3 Å.

After metal binding (∼10 ns), Glu89 flips back into its initial inner conformation, carrying the coordinated metal closer to the 5′ phosphate, at ∼3.25 Å. This metal ion is thus brought into a very similar location compared to MgC in the crystal structure of the prereactive state (PDB ID 5V06). After a few hundred nanoseconds (e.g., ∼200 ns for K^+^ ion), the third metal ion departs spontaneously from the catalytic site. Glu89 then flips again into its outer conformation toward the bulk. These unprompted metal binding and release events, synchronized with the flipping of the Glu89 side chain, were observed multiple times in our extended simulations (Figure S7). This indicates that Glu89 may recruit a third metal ion, bringing it transiently closer to the catalytic site.^46^

To further test Glu89’s role as metal-ion recruiter, we ran multiple MD simulations (∼1 μs in total), inserting the Glu89Ala mutation in the reactant state in the absence of MgC. The overall stability of the enzyme–DNA complex was maintained, with a low RMSD value of 1.27 ± 0.15 Å (see Figure S2). The prereactive state at the active site was also maintained throughout the simulations, that is, the two catalytic metal ions, MgA and MgB, stably maintained their internuclear distance. The nucleophilic water molecule remained properly positioned in front of the scissile phosphodiester bond and the catalytic residues. Finally, Lys85 and Arg92 maintained their initial interaction network with the scissile phosphate. Notably, in this mutated system, we did not see any ion approaching the terminal 5′ phosphate from the bulk solvent. This result is supported by *g*(*r*), which confirms that no ion is located within ∼6 Å of the center of mass of the 5′ phosphate group (Figure 2), further suggesting that Glu89 recruits a third metal ion from the bulk.

### Third Ion Promotes Leaving Group Departure after DNA Hydrolysis

Here, we used MD simulations (∼1 μs in total) of the products of the wild-type (*wt*) native state. Thus, we inserted a native aspartate at the Asp225Ala mutation and replaced Mn with native Mg ions in the postreactive crystal structure (PDB ID 5V0A). Notably, at this catalytic stage, the DNA’s processed strand is enzymatically cleaved, with the consequent generation of the leaving group, i.e., the adenosine 5′-monophosphate (AMP) nucleotide, which is now detached from the newly formed 5′ recessed-end substrate. Importantly, the third ion is not present at the catalytic active site in the crystallographic structure.

**Figure 3:**
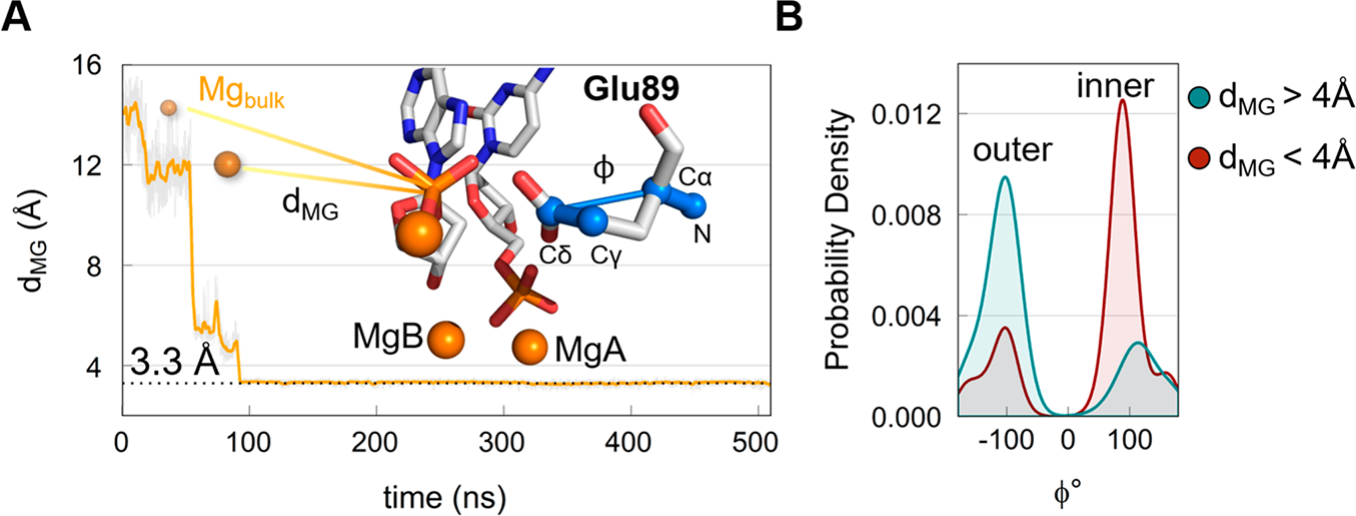
(A) Distance (*d*_MG_ in yellow) between the third Mg^2+^ ion, from the bulk (Mg_bulk_), and the phosphorus of the 5′ phosphate group of AMP. Inset, representation (snapshot from the PS_2M_ simulations) of Mg_bulk_ approaching the terminal 5′ phosphate as well as the pseudo-dihedral angle ϕ of Glu89 side chain (defined by the N–Cα–Cδ−-Cγ atoms). (B) Probability density of the pseudo-dihedral angle ϕ in Glu89 during the simulation: (blue) probability density for *d*_MG_ values > 4 Å shows the outer conformation as the most populated; (red) probability density for d_MG_ values < 4 Å shows the inner conformation is the most populated.

**Figure 4:**
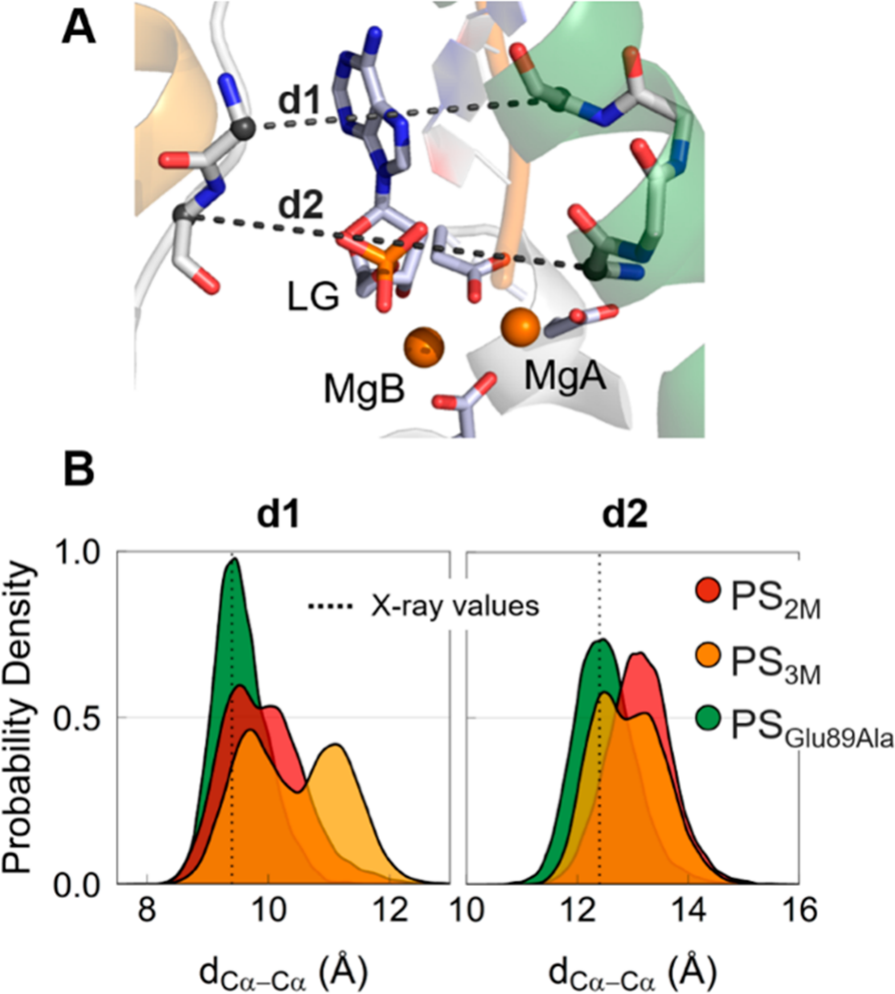
(A) Graphic representation of d1 and d2 distances (PDB ID 5V0A). (B) Probability density of the distances d1 and d2 calculated during simulations of the systems PS_2M_ (in red), PS_3M_ (in orange), and PS_Glu89Ala_ (in green).

In the postreactive crystal structure, the Glu89 side chain adopted an intermediate conformation (ϕ of Glu89 was −36°, Figure S1) between the inner (∼+100°) and the outer (∼−95°) conformations. Then during the MD simulations, the Glu89 side chain stably adopted an outer conformation (ϕ of Glu89 becomes ∼−100°). However, after ∼50 ns, we observed the unprompted approach of a third Mg^2+^ ion from the bulk water (Mg_bulk_), which came close to the Glu89 side chain. This transient ion thus reached a position close to the 5′-monophosphate of the AMP at a distance of ∼3.3 Å, which was equivalent to that in the prereactive simulations and crystal structure (PDB ID 5V06). In this position, the third metal ion interacted with the 5′ phosphate and Glu89 for the remaining simulation time (Figure 3A). During this event, the two catalytic metal ions moved apart slowly, reaching a distance of ∼5.5 Å (compared to 3.9 Å in the starting model PDB ID 5V0A). The drifting of the internuclear two-metal-ion distance was coupled to a shift in the leaving AMP. This is described well by the collective variable CV1, which measures the distance between the center of mass (COM) of the heavy atoms of AMP and the COM of the Cα of the aspartates in the first coordination shell of the two-metal-ion center (i.e., Asp152, Asp171, Asp173) (Figure S8A). During our simulations, CV1 increased by ∼2 Å, from 9 to 11 Å, reflecting the partial exit of the leaving AMP (Figure S8B and S8C). Moreover, His36, which was initially in the down orientation, immediately rotated into the up conformation (Figure S9), forming a π–π interaction with AMP. This interaction helps the initial displacement of the leaving AMP. Notably, the up conformation of His36 was found in the structure of the enzyme after AMP departure (PDB ID 5V0B),^23^ which further suggests the need of this rotation during leaving group release. We also noted a gradual and slight opening in the gateway region at the bottom of the mobile arch, which however maintained an ordered secondary structure (Figure S10). This event is described well by the increase of ∼1 Å of the two distances d1 and d2, which reflect the opening of the α4/α5 interhelix passage (calculated using the Cα of Glu89 and Arg92 along the α4 helix and the Cα of Asn124 and Ile125, located at the bottom of α5 helix; see Figure 4). The probability density function of ϕ, calculated for two states (Mg_bulk_ > 4 Å or Mg_bulk_ < 4 Å from the 5′ phosphate), shows the relative peaks of the two conformations assumed by Glu89, i.e., inner and outer (Figure 3B).

In the products, Arg96 invariantly interacts with the 5′ phosphate in AMP (Figure S6), as also reported for the reactant state simulations in the presence of the MgC. Moreover, after ∼450 ns, we noted a second arginine residue located along the α4 helix, Arg93, which approached the same 5′ phosphate of the AMP, at ∼5 Å. These interactions thus favor the positional shift and partial departure of the AMP from the catalytic site, as indicated by CV1, with consequent destabilization of the two-metal-ion site. We compared these results with an additional postreactive model, which initially contained a third ion at the catalytic site (∼1 μs in total, see SI). These simulations confirmed the enhanced instability of the prone-to-escape leaving group with a partial shift from its starting position during the simulations (Figure S8). Taken together, these results suggest that complete leaving group departure is eventually expected, although this would require longer simulations and would also likely implicate the overtaking of an energetic barrier.^47^

As with the reactant state (see above), we also ran simulations (∼1 μs in total) in the product state of a system with the Glu89Ala mutation in the absence of MgC. The overall stability of the enzyme–DNA complex was maintained (Figure S2). Importantly, in the absence of the Glu89 recruiter, no ion approached the 5′ phosphate of the leaving group, as shown by *g*(*r*) (Figure 2). As a result, the leaving group also showed higher stability in its position, as highlighted by the low RMSD value of 1.71 ± 0.18 Å. Moreover, Arg93 almost never interacted with the 5′-phosphate of AMP, and Arg96 stably maintained its initial interaction with AMP. In addition, we did not observe any opening in the gateway region, with the distances d1 and d2 remaining unchanged during the simulations (Figure 4). These results support the hypothesis that Glu89 recruits MgC before (or during) DNA cleavage. In return, MgC seems to promote the release of the leaving group after the chemical step for phosphodiester bond hydrolysis, acting as a shuttle for the AMP departure.^47^

### Energetics of the Glu89 Flipping and Leaving Group Departure via Metadynamics Simulations

To sample and determine the semiquantitative energetics of the inner ↔ outer conformational switch of Glu89, we used the pseudodihedral angle ϕ as the collective variable to run multiple metadynamics simulations, with and without the third metal ion at the catalytic site, in the reactant and product states (for a total of ∼920 ns).

**Figure 5:**
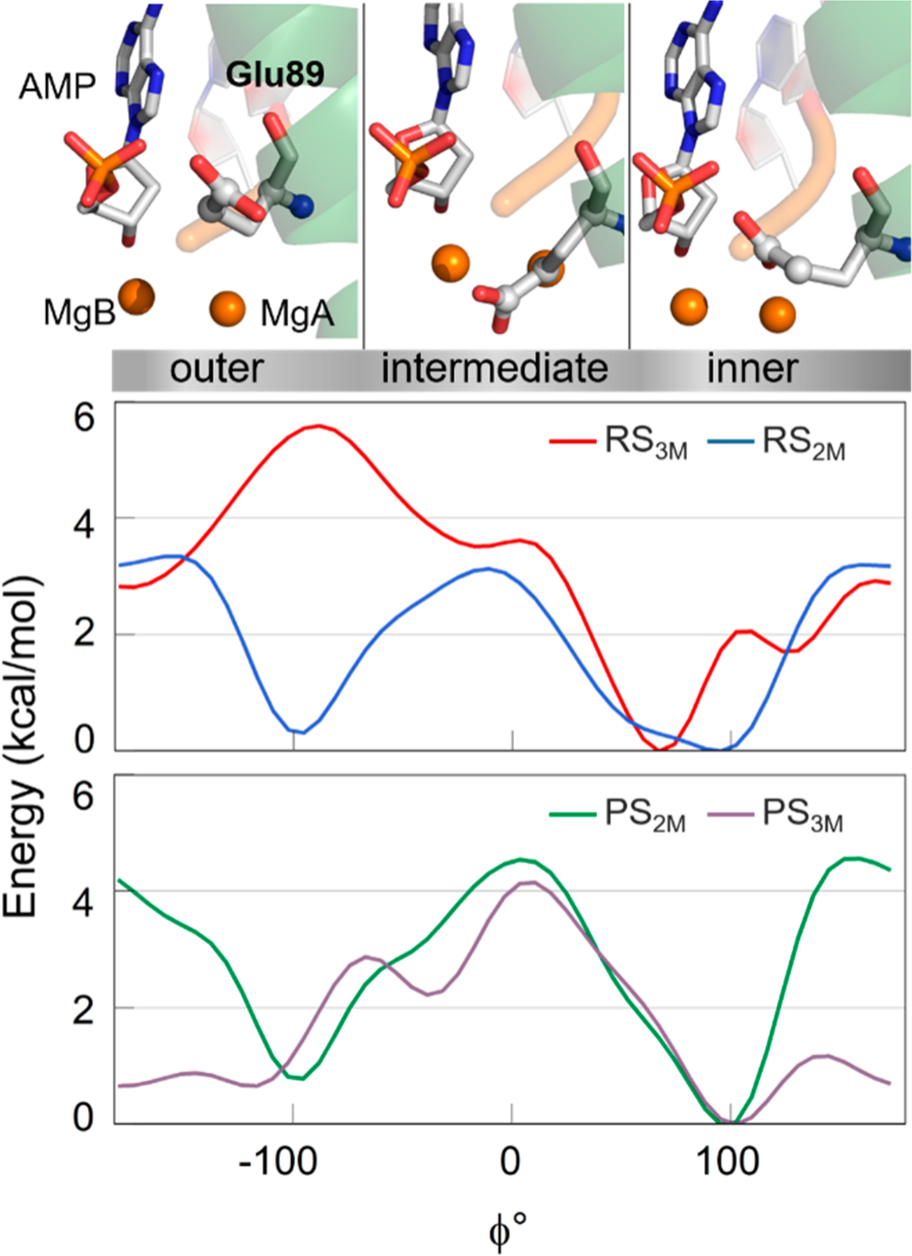
(Bottom) Free energy surface obtained through well-tempered metadynamics simulations for RS_3M_ (red), RS_2M_ (blue), PS_2M_ (green), and PS_3M_ (light purple) systems. Results show three conformations (outer, intermediate, inner). (Top) Graphic representations, taken from PS_3M_ simulaitons, of the three conformations are shown in licorice.

In the reactant state with the third ion, Glu89 tended to adopt inner conformations located in an energy minimum at ϕ ≈ 70°, while outer conformations were not visited due to their high energy (Figure 5, red profile). In the inner conformation, MgC stayed close to the reactive center. However, in the absence of the third metal ion, Glu89 could be found in two isoenergetic minima, i.e., inner and outer conformations, separated by a barrier of only ∼3 kcal mol^–1^ (Figure 5, blue profile). This explains the fact that both Glu89 conformations were similarly populated in our unbiased MD simulations of the system without the third metal ion.

In the product state, regardless of the presence or absence of the third ion, Glu89 visited both the inner and the outer conformations (Figure 5, green and light purple profiles). The conformational switch showed a barrier of ∼4.5 kcal mol^–1^, with or without MgC. We also located a metastable conformation of Glu89 bound to MgC, at ϕ ≈ −40°, in which the glutamate’s side chain adopted an intermediate orientation between the two minima (inner and outer). Interestingly, this metastable state corresponds well to the crystallographic conformation (ϕ = −36°, PDB ID 5V0A) in which MgC is missing. This is likely because, at this point, Glu89 is already solvent exposed.

**Figure 6:**
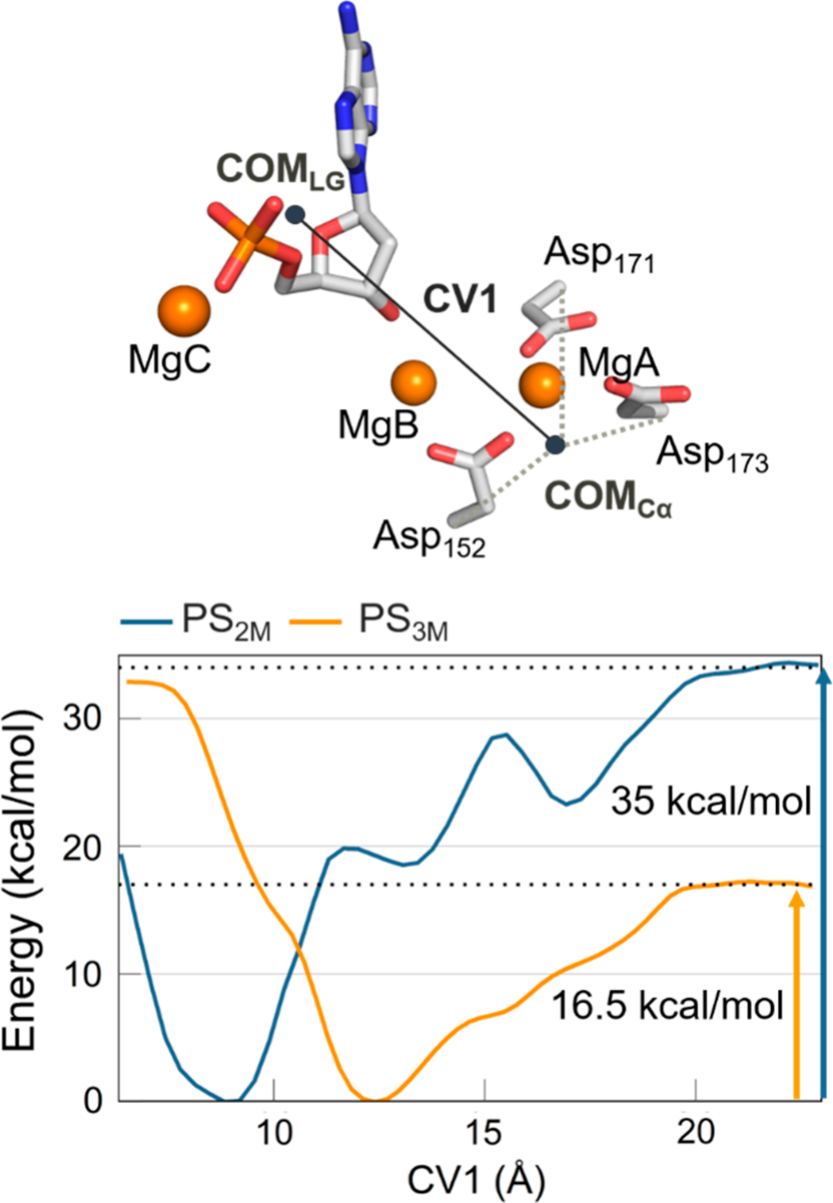
Free energy surface obtained through confined well-tempered metadynamics simulations for PS_2M_ (blue) and PS_3M_ (yellow) systems. (Top) Schematic representation of the CV1, exemplified using a snapshot from PS_3M_ simulations. It represents the distance between the center of mass (COM) of the heavy atoms of the nucleotide leaving group and the COM of the Cα of the aspartates (Asp152, Asp171, Asp173) in the first coordination shell of MgA, MgB. Two different minima, at 8.8 and 12.4 Å, agree with the MD results, in which a partial exit of the leaving group (CV1 ≈ 12.4 Å) was seen only in the presence of MgC (Figure S8).

Then we evaluated possible pathways and energetics for the release of the leaving group from hExo1 in the presence and absence of MgC. We used confined metadynamics,^48^ which enhances the sampling of transversal and often slow degrees of freedom of complex (rare) events, such as the exit of the adenosine monophosphate (AMP) nucleotide from the catalytic site (see SI for further information). Here, the collective variable was CV1 (Figure 6), which captures the degree of departure of the leaving AMP from the reactive site (see definition of CV1, above).

In the presence of MgC, AMP fell into a minimum at CV1 = ∼12 Å, showing that the leaving group is already shifted out of the catalytic site (CV1 = ∼8.5 Å in the uncleaved prereactive state). This is in line with the plain MD simulations, where it was only in the presence of the third metal ion that the leaving group partially exited from the active site, increasing the CV1 value of ∼2 Å, reaching a value of ∼12 Å (Figure S8). At this point, to allow the full departure of the leaving group, the freed AMP stayed complexed with MgC. In this way, the AMP/MgC complex exited from the catalytic site, passing through the aperture under the mobile arch formed only when MgC is present (due to MgC-mediated AMP drifting out from the catalytic center). At this point, the Glu89 sampled the inner and outer conformations until the leaving group overcame the gateway region. At this point, Glu89 stably adopted the inner conformation, in agreement with the crystallographic structure of the complex after release of the leaving group (PDB ID 5V0B). The physical step for AMP/MgC unbinding occurred with a barrier of ∼16.5 kcal/mol (Figure 6).

In the absence of the third ion, the energy barrier for the overall unbinding process was much higher at ∼35 kcal/mol. Indeed, the system behaved quite differently. In the initial configuration, the leaving AMP fell into an energy minimum where it was poorly solvated, at CV1= ∼9 Å (compared to the case where it was solvated and complexed with MgC, CV1= ∼12 Å, Figure 6). From this state, the exit of the AMP alone had to overcome a first barrier of ∼20 kcal/mol, which is already higher than the barrier in the presence of MgC. Then the exit path showed metastable states (relative energy minima at CV1 = ∼13.5 Å and CV1 = ∼17 Å, Figure 6) where the leaving AMP seemed to be transiently trapped by the formation of short-lived interactions with the enzyme. This may slow the AMP unbinding kinetics. Notably, these transient interactions were not formed for the AMP/MgC leaving complex. It is also worth noticing that in both systems PS_3M_ and PS_2M_ we observed the exit of MgB from the catalytic pocket, which occurred concertedly with the exit of the AMP leaving (see Movie in SI). In detail, the MgB catalytic ion remained coordinated to the oxygen of the OH in C3 position of the sugar of the AMP leaving group. Interestingly, the concerted departure of MgB, MgC, and the leaving group AMP agrees with previous studies showing that MgB dissociates from the catalytic site ∼200 times faster compared MgA.^49^ Thus, the concerted exit of both MgB and MgC is found here to promote AMP departure by stabilizing the newly formed negative charge on the leaving group after substrate hydrolysis.^47^

## Discussion

Recently, a time series of structural intermediates captured during human exonuclease1 (hExo1) catalysis has revealed the presence of a third metal ion (MgC) close to the active site.^23^ Intriguingly, a transient metal ion was recently observed in a few DNA/RNA-processing enzymes.^1,2,4,50^ The role of this third additional metal ion at the catalytic center is still unclear.^44^ Here, we used force-field-based molecular dynamics (MD) simulations and free-energy calculations to investigate the recruiting mechanism and functional role of a third metal for hExo1 catalysis. We simulated and compared several model systems built with recent hExo1 structures of the wild-type (*wt*) hExo1/DNA complex in the reactant and product states with and without MgC.

During our multiple and extended MD simulations (∼6 μs in total) we first observed that Glu89 sometimes oscillated but mostly maintained its starting conformation. In this inner conformation, the Glu89 carboxylate group points toward the 5′ phosphate. However, at times and only in the absence of MgC the Glu89 carboxylate group switched its orientation, adopting outer conformations that pointed toward the bulk solvent. Free-energy calculations confirmed that in the reactant state and in the presence of MgC Glu89 tended to adopt inner conformations, which are located in an energy minimum at ϕ ≈ 70°. Outer conformations are not visited due to their high energy. However, in the absence of MgC, the inner and outer conformations become isoenergetic, with a barrier of only ∼3 kcal/mol in between.

Importantly, following this conformational switch in equilibrium MD simulations in the absence of MgC, we observed that transient monovalent ions were freely recruited from the bulk by the outer conformation of Glu89. This conformation therefore seems to act as an anchor point for (third)metal–enzyme complexation. Then Glu89 could switch back and adopt the inner conformation, bringing the bound metal ion (either K^+^ or Na^+^, from these simulations) close to the terminal 5′ phosphate (∼3.5 Å) (Figure S7). This third metal was spontaneously recruited and located in the same position as the third ion captured in the prereactive crystal structure (PDB ID 5V06). This Glu89-mediated mechanism for metal recruitment was further validated by simulations of mutated Glu89Ala systems. These simulations confirmed that in the absence of Glu89 no metal ion from the bulk was spontaneously recruited close to the catalytic center. Interestingly, the role of Glu89 in hExo1 is similar to the role previously proposed for Glu188 in *Bacillus halodurans* ribonuclease H (*Bh*RNase H), where MD simulations suggested that this residue attracted a transient third ion.^46^ Intriguingly, a transient third solvent-exposed cation was found close to the two-metal-ion active site of *D. mobilis* homing endonuclease, I-DmoI.^51^

In the unbiased MD simulations of the product state we observed the unprompted entry of the third metal ion MgC from the bulk, reconstituting the three-metal-ion system (Figure 3). This happened concomitantly to the rotation of the Glu89 side chain from outer to inner, thus destabilizing the geometry of the catalytic active site. Notably, during this process with the double-strand DNA already bound to hExo1, the enzyme maintained an ordered secondary structure of the mobile arch. After the initial DNA binding and subsequent DNA hydrolysis, the enzyme will have to position the “next” scissile bond into the active site. An ordered structure may thus favor the processivity of the exonuclease activity of this enzyme. At this stage the system evolved toward the final catalytic step, i.e., the exit of the leaving group from the catalytic site. In this system, Glu89 was free to populate the inner and outer conformations, overcoming an energy barrier of ∼4.5 kcal/mol, calculated from metadynamics simulations. In addition, we computed the energetics for the full release of the leaving group in the presence or absence of MgC using confined well-tempered metadynamic simulations.^48^ The energetic barrier for AMP departure was ∼16.5 kcal/mol in the presence of MgC and ∼35 kcal/mol in its absence.

The Gibbs free energy (Δ*G*^‡^) for the overall catalytic process of hExo1 is 19.6 kcal/mol, computed using the experimental *k*_cat_ for hExo1 (see SI for further information).^31^ This energy value corresponds fairly well to our estimation of the free-energy barrier for the unbinding process of the leaving group in the presence of the third ion, i.e., ∼16.5 kcal/mol. The leaving group departure may therefore be rate limiting for the exonuclease catalytic process in hExo1, as already proposed for other metallonucleases (e.g., FENs, APE1, PvuII, *Mun*I, *Nae*I, *Sfi*I, *Eco*RI, *Eco*RV).^52−59^

These results suggest a mechanism where Glu89 recruits a third metal ion in the reactant state. Clearly, quantum calculations are needed to evaluate the mechanistic implications of this additional ion for the chemical step of DNA hydrolysis.^60−63^ However, from these classical MD simulations, it emerges that the third ion promotes leaving group departure, acting as a shuttle for the exit of the nucleotide monophosphate product from the catalytic site. Notably, this result is in line with evidence of a third-ion-mediated leaving mechanism for pyrophosphate departure in polymerase enzymes.^47^

To further test this mechanistic hypothesis and investigate whether this enzymatic strategy is shared by other nucleases, we performed sequence alignments via the Needleman–Wunsch algorithm^64^ using 10 different eukaryotic species of Exo1 (see SI for more information). We found that the Glu89 is fully conserved among these enzymes, as for those residues forming the reaction center, and second-shell residues like Lys85 and Arg92, and the guide residues Tyr32 and His36 (Figure S11). This suggests that Glu89 is an integral part of the enzymatic machinery for efficient catalysis in Exo1.

**Figure 7:**
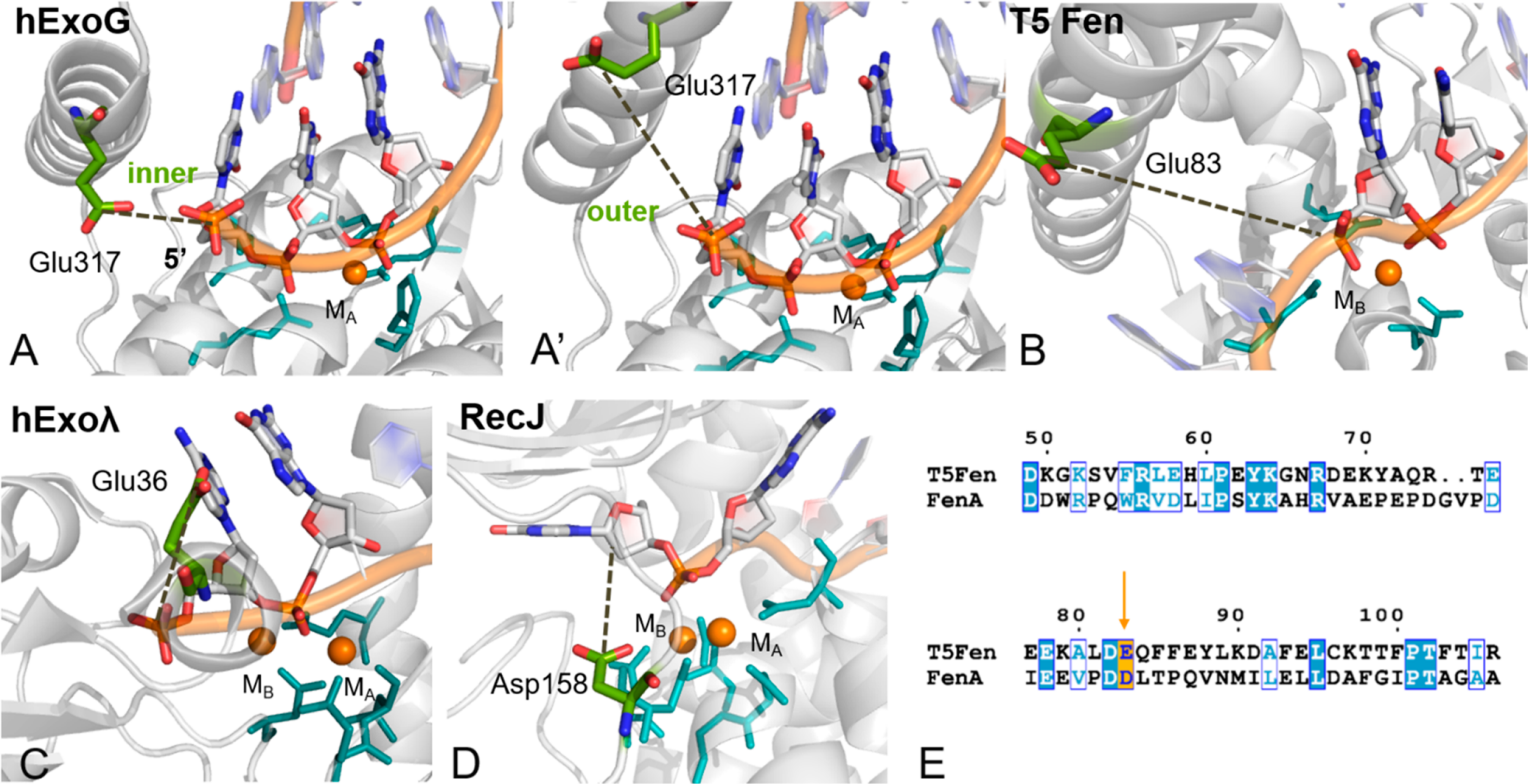
Close views of the active site of 5′ metallonuclease members that possess an analogous acid residue (light green) close to the two-metal-ion center (M_A_, M_B_, in orange), the active site (in cyan), and the leaving group (indicated by a dashed line). (A) *Human* ExoG, in which Glu317 is pointing in the inner (PDB ID 5T5C) and (A′) outer conformations (PDB ID 5T40, merged with the DNA substrate from PDB ID 5T5C). (B) *Escherichia phage* T5Fen (PDB ID 5HNK). (C) *Human* λ-Exonuclease X (PDB ID 3SM4). (D) *D. radiodurans* RecJ (PDB ID 5F55). (E) Sequence alignment of *M. smegmatis* FenA and *E. phage* T5Fen. Conserved acid residue (Glu/Asp) is indicated in orange.

We also performed structural comparisons using recent crystallographic structures of additional nucleases and identified a shared spatial localization in these enzymes of an acidic residue (Glu/Asp), in analogy to Glu89 in hExo1. In such enzymes, in fact, we always identified the presence of a Glu/Asp residue located in a second-shell sphere cantered on the two-metal-ion active site. This acidic residue is always situated in a solvent-accessible position (thus able to recruit ions from the bulk), being strategically located on the side of the expected exit path for leaving group departure, with respect to the catalytic center. For example, *human* ExoG,^65,66^ which is 5′ metallo-exonuclease enzyme cocrystallized in complex with the DNA substrate, has a glutamate (Glu317) residue located near the terminal 5′ phosphate. Here, Glu317 resides in a solvent-exposed area. Notably, Glu317 can assume different orientations in the available crystals (see PDB ID 5T5C vs PDB ID 5T40),^65^ which suggests that this glutamate may act as a recruiter of metal ions in the same way as for Glu89 in hExo1 (Figure 7A). Another case is the *human* λ-Exonuclease,^67,68^ where Glu36 is located close to the 5′ phosphate of the DNA substrate (Figure 7B). Here, too, it has been hypothesized that a third metal ion may transiently bind close to the two-metal-ion site, likely aiding the leaving group departure.^49^ A further example is RecJ nuclease,^69^ where Asp158 is solvent exposed and close to the active site in a similar position as Glu89 in hExo1 (Figure 7C).

Then we looked at the hExo1 family member bacteriophage T5 flap endonuclease (T5 Fen, PDB ID 5HNK).^16^ We identified Glu83, which is located along the mobile arch, in analogy to Glu89 in hExo1. Finally, we considered the recent high-resolution X-ray structure of *M. smegmatis* FenA,^17^ which is a 5′ structure-specific nuclease and close homologue of phage T5Fen, and its sequence alignment with T5Fen. It suggests that Glu83 in T5Fen may correspond to Asp85 in FenA (Figure 7D and 7E). Notably, holo forms of T5Fen and FenA were crystallized in complex with three metal ions in the active site.^16,17^ This further corroborates the idea that multimetal-ion catalytic sites may be necessary for nucleic-acid processing in these enzymes. Indeed, while the presence of a third metal ion, located in the vicinity of the two-metal-ion active site, is a novel aspect in polymerases and nucleases, the exact position of such additional metal ion with respect to the catalytic center can vary.^4^ For example, T5FEN and FenA enzymes were solved with a third ion in a different relative position, although always in close proximity of the reactive two-metal-ion center. It is thus plausible that the third transient ion may play different roles during catalysis, according to its specific location at the catalytic center.

Another intriguing aspect is the recurring presence of second-shell positively charged residues that surround the metal-aided catalytic site in nucleic-acid-processing enzyme.^3^ During our extended MD simulations, we observed the interaction of Arg93, along the helical arch, with the terminal phosphate of the substrate. We structurally aligned hExo1 (PDB ID 5V0E)^23^ with hFEN1 (PDB ID 5KSE),^34^ and we noted that the terminal guanidine, amide, and amine groups of Arg93, Asn124 (in hExo1), and Lys132 (in hFEN1) residues were all within a sphere of ∼3 Å and close to the 5′ phosphate in the leaving group (Figure S12). This result further suggests that Arg93, together with Arg96, may have a crucial role in 5′ phosphate steering.^34^ In this respect, Arg93 is an important point of control in Exo1 poly(ADP-ribose) binding, as proved by in vitro and in vivo assays on natural Arg93Gly mutation.^70^ Moreover, a common feature of different nucleic-acid-processing enzymes is a solvent-exposed and positively charged residue that interacts with the negatively charged moiety in the leaving group (e.g., Arg96–5′ phosphate).^71^ Interestingly, this Arg96–5′ phosphate interaction comes in addition to an already complex architecture characterized by a number of positively charged residues surrounding the active site. Indeed, hExo1 is one of a large set of nucleic-acid-processing enzymes characterized by the recurring presence of positively charged elements in the vicinity of the reactive site.^3^ These elements in hExo1 comprise the catalytic Lys85 and Arg92. These positively charged residues are thought to play a role in the phosphate steering during the threading mechanism. These residues therefore seem crucial for substrate recognition, binding, catalysis, translocation, and initial product release. However, the Glu89-mediated recruitment and binding of a third ion from the bulk also seems necessary for the full departure of the leaving group from the enzyme’s catalytic site in hExo1 and likely in several other nuclease enzymes.

## Conclusions

Our results provide new insights into the functional role of a third metal ion, which was recently found transiently located at the catalytic site of nuclease enzymes during catalysis. Using molecular dynamics and free-energy simulations applied to multiple systems, we considered the conformational switch of the side chain of a specific residue, Glu89, which is located near the active site in hExo1. We noted that this conformational switch favors the recruitment of a third metal ion from the bulk. The third metal ion is thus promptly positioned near the catalytic center, in accordance with the structural evidence. Our simulations also indicate that this ion serves as an exit shuttle for the leaving group departure from the catalytic site after DNA hydrolysis. The exit mechanism is also favored by the initial involvement of positively charged residues, which are located in an extended and highly structured second-shell area at the two-metal-ion active site.^3,72,73^ Finally, our structural analyses of nuclease enzymes show that such a negatively charged residue (Glu/Asp) is persistently found in a similar, structurally conserved, and strategic position in several other 5′ structure-specific nucleases, which seem to share this enzymatic mechanism to promote DNA hydrolysis. These findings may have an implication for de novo enzyme engineering and structure-based drug design.^74,75^

## Methods

### Structural Models

We used six different model systems: (i) the wild-type (*wt*) reactant state (RS_3M_), based on the recent time-resolved X-ray structure of the complex of the hExo1(PDB ID 5V06), which includes the 5′ recessed-end DNA substrate (in this structure, the third metal ion is located close to the catalytic site); (ii) the same *wt* reactant state with the third metal ion manually removed (RS_2M_); (iii) the mutated Glu89Ala reactant state (RS_Glu89Ala_), modeled on the same X-ray structure (PDB ID 5V06); (iv) the product state (PS_2M_), based on the recent time-resolved X-ray structure of the ternary complex of hExo1(PDB ID 5V0A), characterized by the newly formed 5′ recessed-end DNA substrate and the leaving adenosine monophosphate (AMP); (v) the product state with the third metal ion close to the 5′ phosphate of the AMP (PS_3M_) (this system is modeled on the X-ray structure of the prereactive complex, in which we manually cleaved the scissile phosphate); (vi) the mutated Glu89Ala product state (PS_Glu89Ala_), modeled on the same X-ray structure (PDB ID 5V0A).^23^ Protein coordinates and relevant trajectory files are available from the authors upon request.

### Classical Molecular Dynamics Simulations

To investigate the functional dynamics of the hExo1/DNA complex, we used extensive force-field-based MD simulations, which are highly informative for complex enzyme/nucleic acid assemblies.^76−80^ Here, the AMBER/ff14SB^81^ and OL15^82,83^ force fields were used to treat the hExo1 enzyme and the DNA, respectively. The terminal 5′ thymine monophosphate, the 5′ adenosine monophosphate, and the terminal 5′ guanidine monophosphate were treated with the general Amber force field (GAFF).^84^ The atomic charges were derived by fitting the electrostatic potential according to the Merz–Singh–Kollman scheme,^85^ the RESP fitting procedure.^86^ The length of all bonds involving hydrogen atoms was constrained to the equilibrium using the P-LINCS algorithm,^87^ and a time integration step of 2 fs was used. All simulations were performed using GROMACS 5.1 code.^88^ Long-range electrostatic interactions were calculated with the particle mesh Ewald method with a Fourier grid spacing of 1.6 Å.^89,90^ Periodic boundary conditions in the three directions of Cartesian space were applied. The magnesium ions were treated with a nonbonded approach based on the “atoms in molecules” theory partitioning scheme.^91,92^ The systems were solvated with TIP3P water molecules^93^ and neutralized adding Mg^2+^, Na^+^, K^+^, and Cl^–^ ions, as indicated in the crystallization procedure.^23^ The total number of atoms was ∼60 000 for each system (see SI for more information). We followed a two-step procedure for the MD simulations: first, the equilibration phase in which we followed different procedures depending on the system (see SI). Then the production phase was carried out in an NPT ensemble, a constant temperature of 310 K imposed using the velocity-rescaling thermostat,^94^ and a constant pressure of 1 bar maintained with a Parrinello–Rahman barostat.^95^ We collected MD simulations of ∼0.7 μs for RS_3M_ and ∼1 μs for each of RS_2M_, RS_Glu89Ala_, PS_2M_, PS_3M_, and PS_Glu89Ala_ for a total of ∼6 μs of MD.

### Free-Energy Calculations

We used well-tempered metadynamics^96^ to characterize and estimate the free-energy landscape of Glu89 conformational flexibility. We selected a collective variable (CV) that distinguished between the inner, the outer, and the intermediate conformations adopted by Glu89 during the MD simulations. Thus, the selected CV was the pseudodihedral angle ϕ defined by the N, Cα, Cδ, and Cγ atoms on Glu89 (see SI). In particular, based on our MD simulations, inner conformations are adopted at ∼70° < ϕ < ∼100°, intermediate conformations at ∼−40° < ϕ < ∼−10°, and outer conformations at ∼−150° < ϕ < ∼−100°. We performed well-tempered metadynamics by biasing the CV using an initial hill height of 0.02 kcal mol^–1^, a hill width of 0.35 rad, a fictitious CV temperature of 1550 K, and a deposition rate of 1 ps^–1^. The simulations were conducted until convergence (see SI for more information).

Well-tempered metadynamics was also used to evaluate possible pathways and the semiquantitative energetics for the release of the leaving group (i.e., adenosine 5′-monophosphate, AMP) from the active site. We used a confined metadynamics approach,^48^ which excludes regions of the conformational space that are not relevant to the chemical event under investigation. The selected CV was the distance between the center of mass (COM) of the heavy atoms of AMP and the COM of the Cα of the aspartates (Asp152, Asp171, Asp173) in the first coordination shell of the two catalytic metal ions (see SI). This CV indicates the degree of departure of AMP from the active site in our MD simulations. We used an initial hill height of 0.29 kcal mol^–1^, a hill width of 0.6 Å, a fictitious CV temperature of 3720 K, and a deposition rate of 1 ps^–1^. The simulations were conducted until convergence (see SI for more information).

## Supporting Information Available

Setup protocols of the force-field-based MD simulations; details on enhanced sampling free energy calculations and additional analyses; PDB analyses, structure, and sequence alignments (PDF)
Illustrative movie of the overall catalytic process of hExo1, created by merging PDB structures with fragments of trajectories of equilibrium and metadynamics simulations (MP4)
