# Supplementary material for: Recruiting Mechanism and Functional Role of a Third Metal Ion in the Enzymatic Activity of 5′ Structure-Specific Nucleases
Source: J Am Chem Soc. 2020 Jan 15;142(6):2823–34. doi: 10.1021/jacs.9b10656 (PMC7993637; doi:10.1021/jacs.9b10656)
Supplement: Supplementary file 1 — ja9b10656_si_001.pdf [file ja9b10656_si_001.pdf]

Recruiting mechanism and functional role of a third metal ion in the enzymatic activity of 5′ structure-specific nucleases

Elisa Donati[1], Vito Genna[1], Marco De Vivo[1]*

[1]Laboratory of Molecular Modelling & Drug Discovery, Istituto Italiano di Tecnologia, Via Morego 30, 16163, Genoa, Italy.

*Corresponding Authors

Dr. Marco De Vivo

E-mail: marco.devivo@iit.it

Supporting information

Table of Contents

Supplementary Text

Structural Models S3

Classical Molecular Dynamics Simulations S4

Confined Well-tempered Metadynamics S4

Transition State Theory S5

Supplementary Figures

Figure S1. Electron density map of pre-reactive and post reactive states S6

Figure S2. Time evolution of root mean square deviation (RMSD) S7

Figure S3. Graphs reporting stability of the active site S8

Figure S4. Graph representing the distance between Lys85 and the scissile phosphate S10

Figure S5. Graphs representing the coordination number of the MgC and the pseudo dihedral angle (ϕ) S11

Figure S6. Graphs representing the distance between Arg96 and the terminal 5′ phosphate S12

Figure S7. Graph representing the distance between ion from the solvent and the 5′ phosphate concomitant with the inner/outer flipping of Glu89 .... S13

Figure S8. Time evolution of CV1 .... S14

Figure S9. Time evolution of dihedral angle (δ) for H36 .... S16

Figure S10. Time evolution of root mean square deviation for the mobile arch S17

Figure S11. Sequence alignments of Exonuclease1 .... S18

Figure S12. Structure alignment of the hExo1 with hFEN1 .... S19

Figure S13. Graphs representing convergence of metadynamics simulations S20

Figure S14. Population of rotamers (χ1, χ2, χ3) of Glu89 .... S21

Movie S15. Illustrative movie of the overall catalytic process of hExo1, formed by merging PDB structures, MD and metadynamics trajectories S22

Table S16. Description of all the simulations performed S23

Reference S24

Structural Models. To model the reactant state, we employed the X-ray structure of the wild-type pre-reactive system (PDB ID 5V06)[1] with a resolution of 2.75 Å. Notably, we removed the last three residues (residues 355, 356 and 357) due to the fact the previous 8 residues, i.e. residues between 347 and 354, were missing from the X-ray structure. We removed also the residue 346, in order to make the systems consistent with the product system, where the residue 346 is missing. Moreover, also the first residue (Met1) was missing. To reproduce the wild-type structure of hExo1 in a competent state for catalysis, the manganese ions were replaced with magnesium ions. The final structure of the wild-type system includes residues from 2 to 346, and 4 metal ions, the two catalytic MgA and MgB, the third ion MgC and the K^+ bound to the DNA and H2TH motif. In order to model the reactant system without the presence of the third MgC in the vicinity of the terminal 5′ phosphate, we manually re-moved MgC.

From the reactant structure without the third metal ion in the vicinity of the active site, we modelled the Glu89Ala mutant system, where the native Glu89 was replaced by an alanine.

To model the product state, we employed the X-ray structure of the cleaved system (PDB ID 5V0A)[1], with a resolution of 2.38 Å. We removed the last two residues, i.e. residues 355 and 356. Also, in this structure the first residue (Met1) was missing. To reproduce the wild-type structure of hExo1 in the product state, the Ala225 mutation and the manganese ions were replaced with the native aspartate and magnesium ions. As for the reactant mutant system, we built the Glu89Ala mutant for the product system. From the wild-type product structure, the Glu89 was replaced by the alanine.

Then, we modelled an additional product state, which was built using the pre-reactive crystal structure as a template, which also contains the third ion. In this model, we manually cleaved the scissile phosphate in order to free the leaving group, and we inserted a bond between the electrophilic phosphorous and the nucleophilic oxygen. Finally, we also adjusted the geometry of the cleaved newly formed 5′ phosphate, in order to have the correct geometry for a SN_2-like reaction, as expected for this two-metal-ion catalysis. As for the reactant system, the manganese ions were replaced with magnesium ions.

Hydrogen atoms were added to each system. Subsequently, each system was immersed in a water box where the distance between the solute and the edge of the box was set to 12 Å. To neutralize the overall charge of the systems Cl^-, K^+, Na^+ and Mg^{2+} ions have been added. For all these three procedures, we used the tLEAP program of the AMBER 17 package[2].

Classical Molecular Dynamics Simulations. The protocol we adopted to run classical MD simulations was formed by two steps: i) equilibration phase; ii) production phase. For the equilibration phase, each system for the reactant state was minimized using a steepest-descent minimization algorithm keeping everything but the water fixed in the crystallographic position with a harmonic force constant of 5000 kJ mol^{-1} nm^{-2}. Then a second minimization with the same algorithm without any restraint. This procedure was adopted for the reactant state because one water molecule was missing in the first coordination shell of one of the two catalytic metal ions. For the product state the system was minimized using a steepest-descent minimization algorithm. Then, each minimized system was heated from 0 to 310 K in 450 ps in NVT ensemble, keeping the backbone atoms and the two catalytic magnesium atoms fixed in their original positions, using a harmonic force constant of 5000 kJ mol^{-1} nm^{-2}. After this, 5 ns NPT simulation was carried out at 310 K and 1 bar, maintaining backbone and magnesium atoms constrained. Eventually, for the production phase, MD was carried out in the NPT ensemble without any restraint. The first 50 ns of the production phase was considered as part of the equilibration and for this reason it was discarded from subsequent analyses. A short-range neighbour list cut-off of 12 Å was used.[3] A Parrinello-Rahman barostat[4] and a velocity-rescaling thermostat[5] were employed with a relaxation time τ of 2 ps and 0.1 ps, respectively. All the snapshots were saved every 50 ps.

Confined Well-tempered Metadynamics. In the present work, we were interested to calculate the free-energy surface (FES) for the release of the leaving group (AMP) in presence/absence of the third metal ion. In this case, regions of the solvent box very far from the active site were of no interest. Thus, our goal was to sample as much as possible the region where the distance between the centre of mass (COM) of the heavy atoms of AMP and the COM of the Cα of the aspartates in the first coordination shell of the two catalytic metal ions, was no longer than 26 Å. In order to avoid sampling conformational space of no interest, we used a the metadynamics approach, with a confined procedure[6] already applied by our group.[6] This method is based on the confinement of the single walker metadynamics run in a specific region of the conformational space. During the well-tempered metadynamics simulations, the value of the Collective Variable (CV) is checked. If the CV value is lower than a specified CV boundary value, determining the region of interest, then that conformation is stored in a pool of frames. When the CV value is higher, the simulation restarts from a randomly chosen conformation among the stored (see more details in Ref.[6]).

Transition State Theory. The Eyring-Polanyi equation is used to derive the rate of a chemical reaction, from the state A to the state B ($k_{A \to B}$), within the temperature T.[7] The equation derives from the Transition State Theory and has the following formula:

$$k_{A \to B} = \kappa \frac{k_B T}{h} e^{-\frac{\Delta^{\ddagger} G_{A \to B}}{RT}}$$

where k_B is the Boltzmann's constant, h is the Planck's constant, R is the gas constant, $\Delta^{\ddagger} G_{A \to B}$ is the Gibbs energy of activation and κ is the transmission coefficient that is often assumed equal to one in order to refer to the fundamental no-recrossing assumption of Transition State Theory. The Transition State Theory provides a simplistic view of the kinetic of the enzyme and here it is used to compare the order of magnitude of the enzymatic turnover rate. From the experimental value of the catalyst rate constant, k_{cat},[8] we derived the theoretical value for the Gibbs energy of activation for the rate-determining step of the overall catalytic process using the Eyring equation. The result obtained agrees with our estimation of the free energy barrier for the leaving group departure process, which is therefore compatible with the overall catalytic reaction.

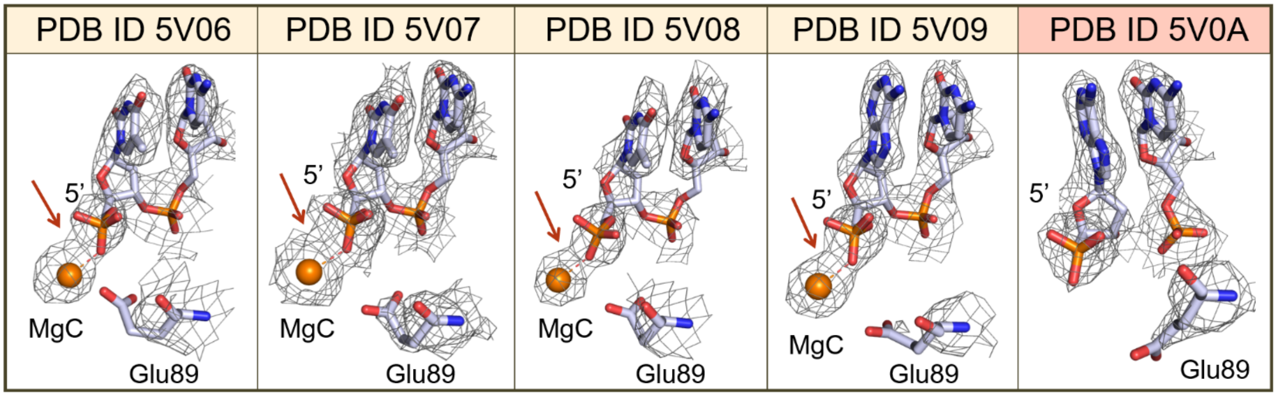

Figure S1. Simulated annealing omit map (1σ contour) identifies the presence of M_C coordinated by the terminal 5′ phosphate (PDB ID 5V06, 5V07, 5V08, 5V09) and the different orientations of Glu89 in the pre-reactive and post-reactive states. Diffraction data were collected at 100 K at the Advanced Photon Source or the Advanced Light Source, as reported in Shi, Y. et al (Ref. 1 in SI).

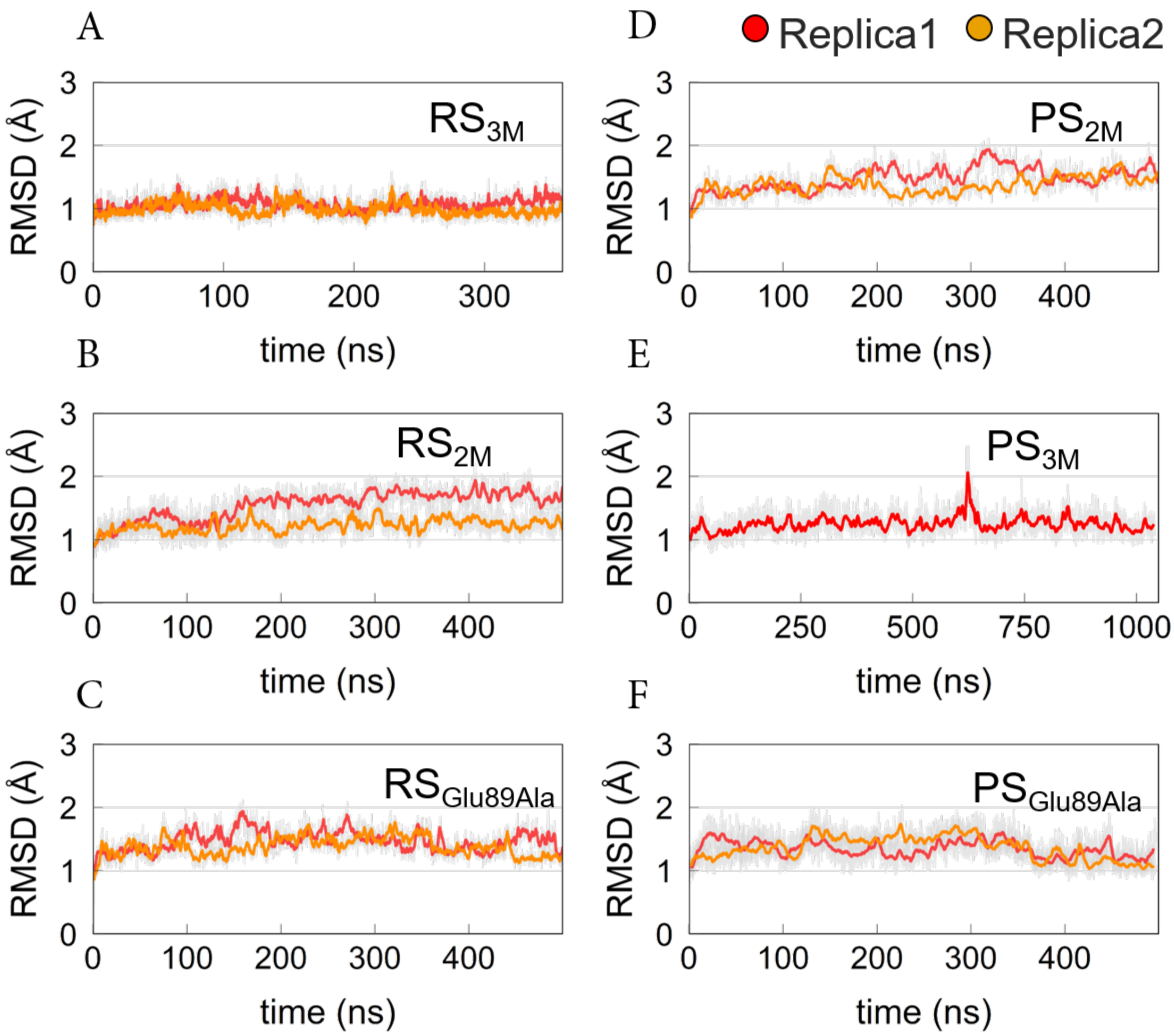

Figure S2. Time evolution of root mean square deviation (RMSD) for the backbone atoms of hExo1. (A) Wild-type Reactant State (RS_{3M}). The average RMSD value for the Replica1 is 1.02 ± 0.11 Å and for Replica2 is 0.99 ± 0.12 Å Å. (B) Wild-type Reactant State without the third metal ion (RS_{2M}). The average RMSD value for the Replica1 is 1.56 ± 0.23 Å and for Replica2 is 1.23 ± 0.14 Å. (C) Mutated Glu89Ala Reactant State ($RS_{Glu89Ala}$). The average RMSD value for the Replica1 is 1.27 ± 0.15 Å and for Replica2 is 1.17 ± 0.15 Å. (D) Wild-type Product State (PS_{2M}). The average RMSD value for the Replica1 is 1.46 ± 0.22 Å and for Replica2 is 1.46 ± 0.17 Å. (E) The Product State with the third metal ion (PS_{3M}). The average RMSD value is 1.25 ± 0.15 Å. (F) Mutated Glu89Ala Product State ($PS_{Glu89Ala}$). The average RMSD value for the Replica1 is 1.35 ± 0.17 Å and for Replica2 is1.35 ± 0.21 Å**.**

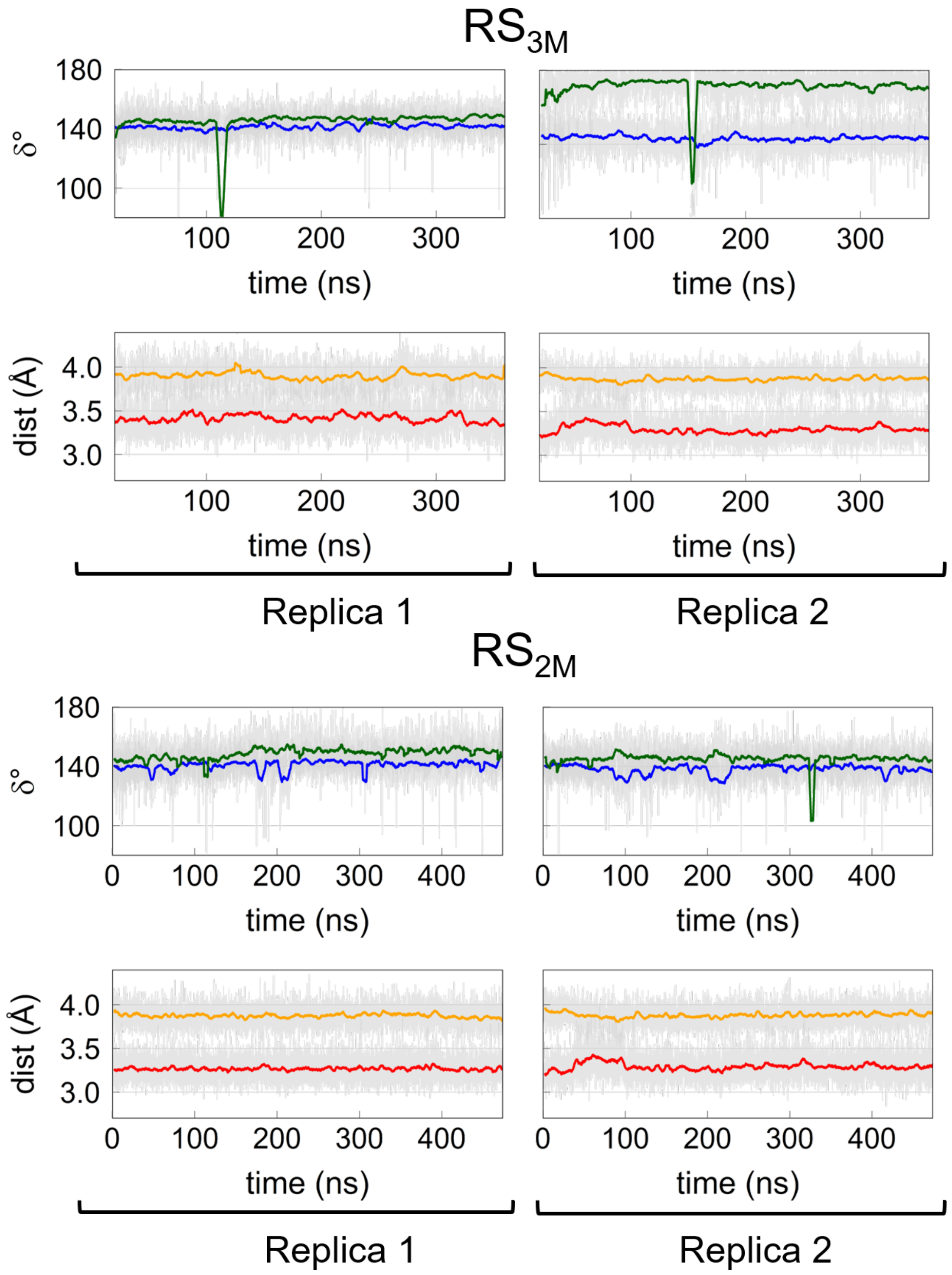

Figure S3. Continue on the next page

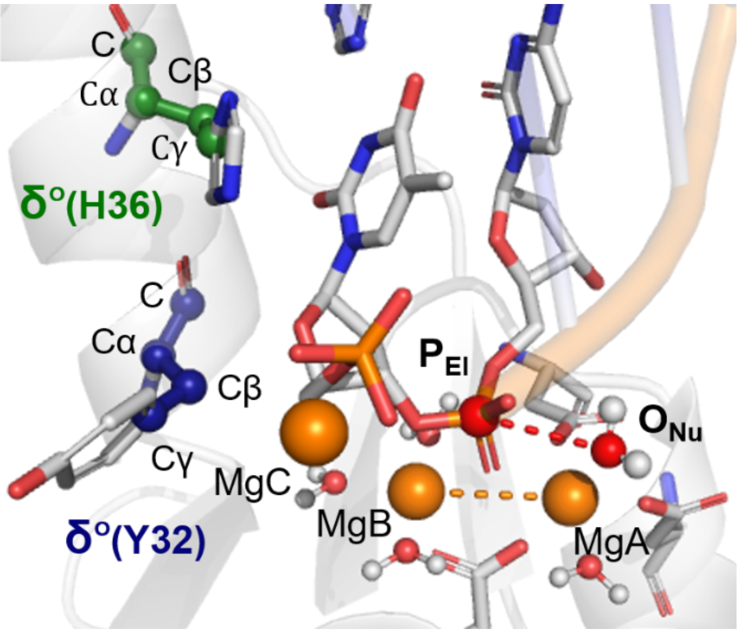

Figure S3. (A) Representation of time evolution distances between: i) the nucleophile oxygen of the water (O_{Nu}) and the phosphorous (P_{EI}), in red and between ii) the two catalytic magnesium ions (MgA, MgB), in orange. Representation of time evolution of the dihedral angles (δ), taken along C, Cα, Cβ, Cγ bonds, for the guide residues Tyr32, in blue, and His36, in green. Data are taken from the reactant systems RS_{3M} and RS_{2M}. (B) Schematic representation of the distances and dihedral angles reported in (A). The structure is taken from PDB ID 5V06.

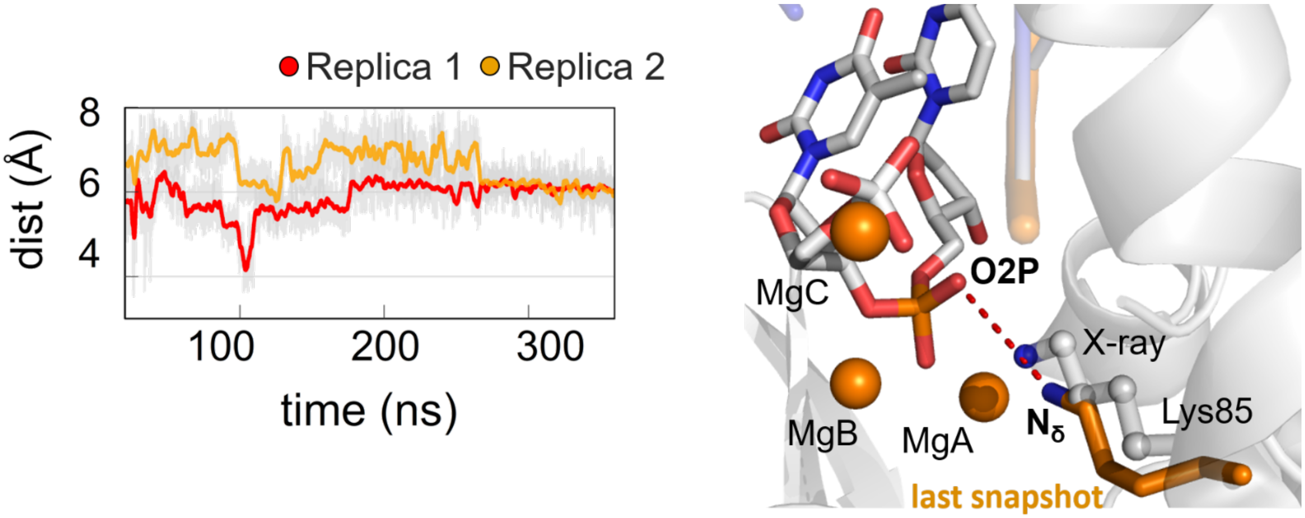

Figure S4. (left) Representation of time evolution distance between nitrogen atom of the terminal amine group of Lys85 (N_δ) and oxygen atom of the scissile phosphate (O2P). (right) Schematic representation of the distance analysed. Lys85 in the last snapshot of the simulation is represented in licorice (orange); the magnesium ions, the DNA nucleotides and Lys85, in the crystallographic position (PDB ID 5V06) are represented in licorice and ball and stick, respectively (white).

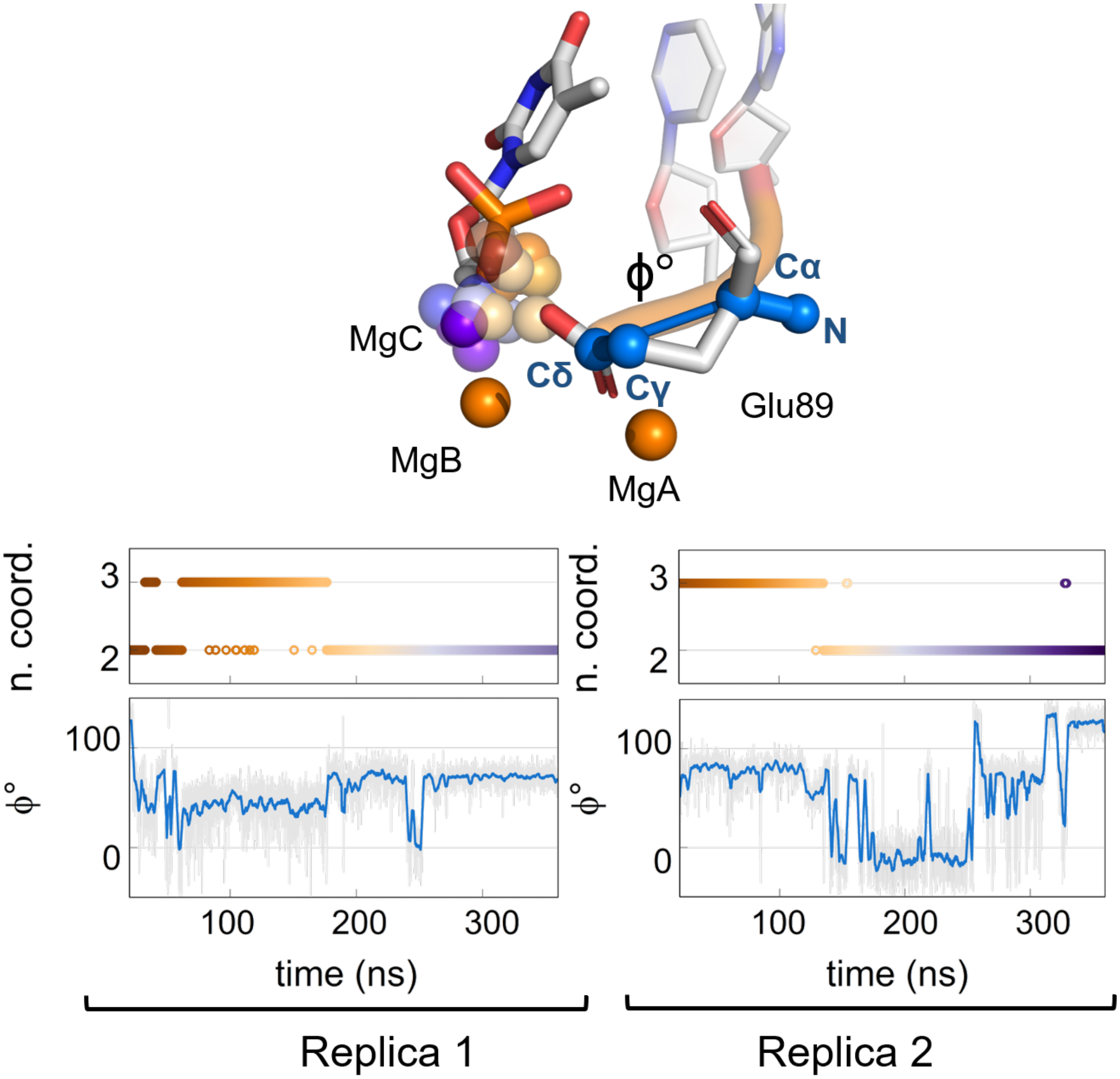

Figure S5. (Bottom) Graphs of time evolution of coordination number of the MgC ion with non-water oxygen atoms (coloured from brown to purple, as a function of time) and the pseudo dihedral angle (φ) taken along N, Cα, Cδ, Cγ bonds of Glu89. The octahedral geometry of MgC is maintained for the entire simulation time and it is completed by water molecules. (Top) Schematic representation of the pseudo dihedral angle (φ) and the different positions of MgC during time, exemplified using a snapshot taken from RS_{3M} simulations (the colour scheme for the MgC spheres is the same used for the coordination number in the plots in the bottom).

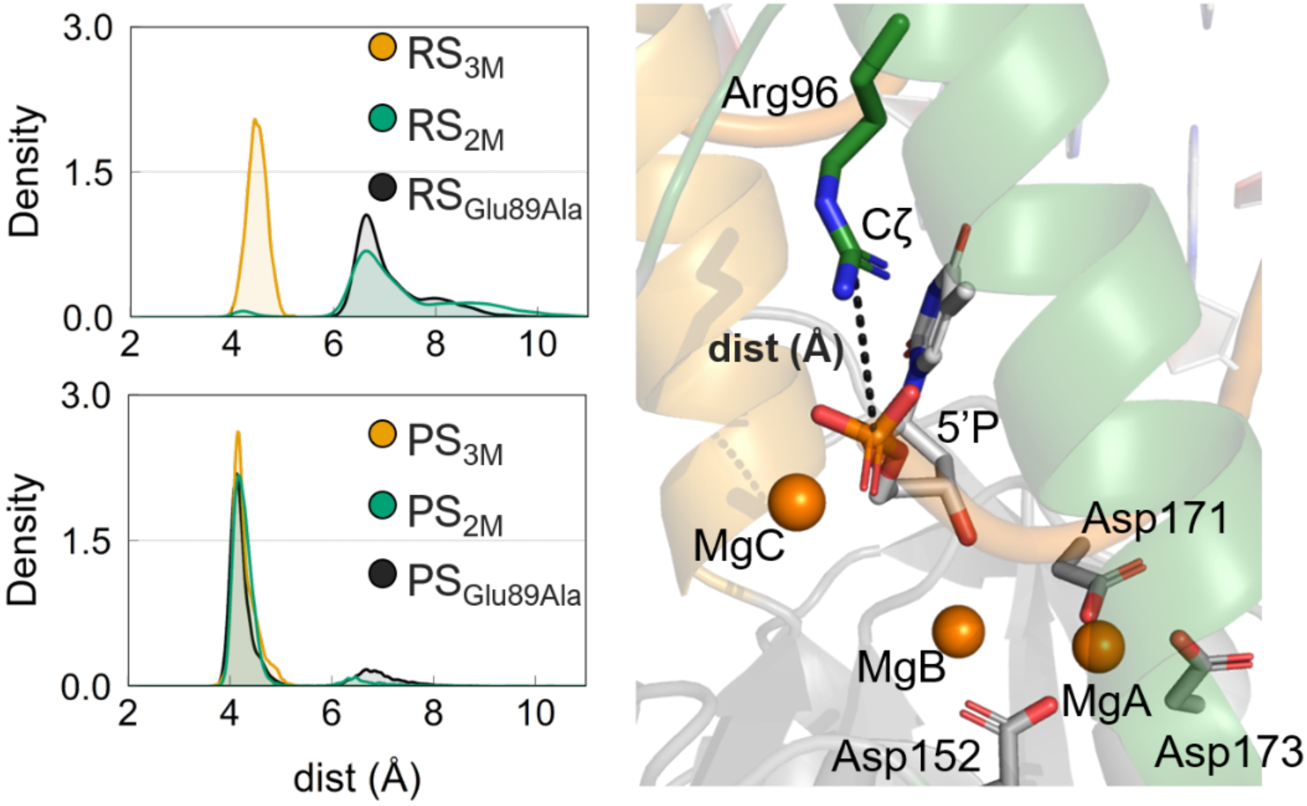

Figure S6. (left) Frequency distribution of the distance between Cζ in Arg96 and phosphorous in the terminal 5′ phosphate group. Top, the frequency distribution related to the Reactant State; Bottom, the frequency distribution related to the Product State. (right) Schematic representation of the terminal 5′ nucleotide, the Arg96 and the catalytic site, taken from RS_{3M} trajectory.

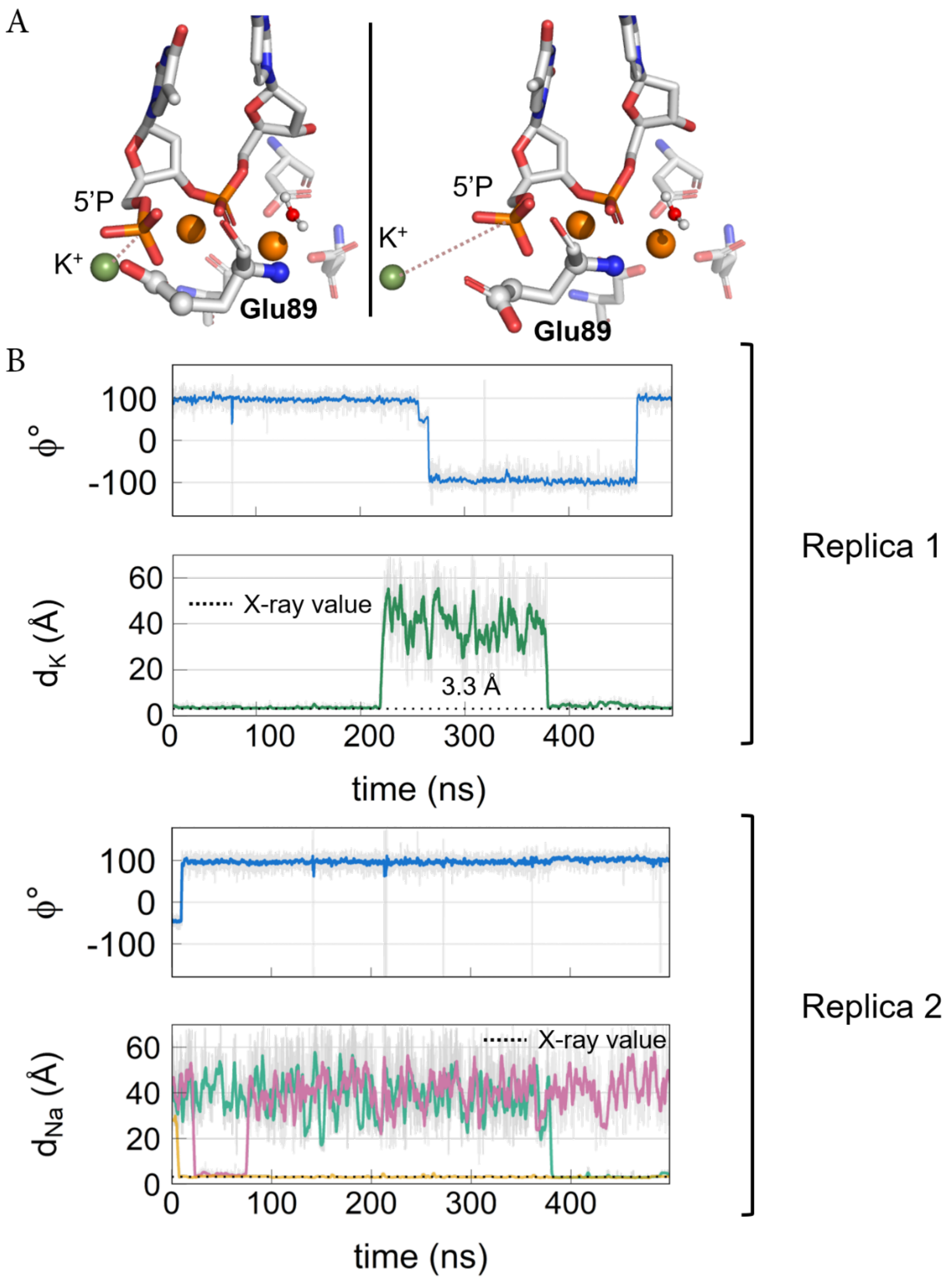

Figure S7. (A) Schematic representation of the distance between K^+ ion from the solvent bulk and the terminal 5′ phosphorous concomitant with the inner/outer flipping observed during the RS_{2M} (Replica1) trajectory. (B) Graphs of time evolution of the pseudo dihedral angle (φ) taken along N, Cα, Cδ, Cγ bonds of Glu89, which describes the inner and the outer conformations and the representation of time evolution of the distance between the monovalent ion from the solvent bulk and the terminal 5′ phosphorous. Data are taken from the RS_{2M} system.

A

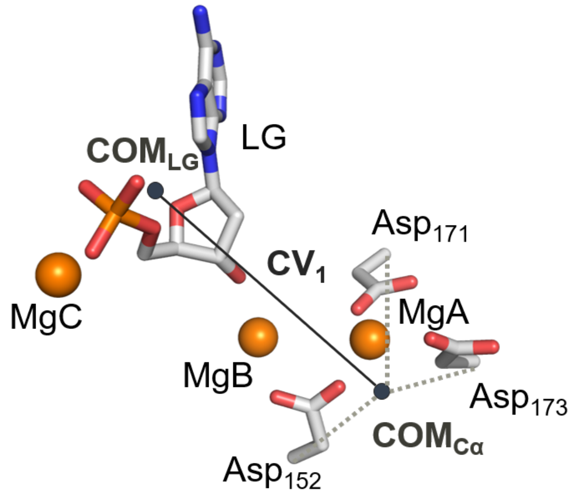

B

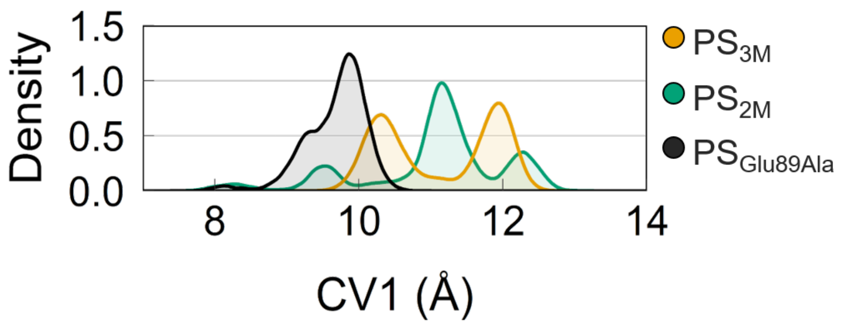

Figure S8. Continue on the next page.

C

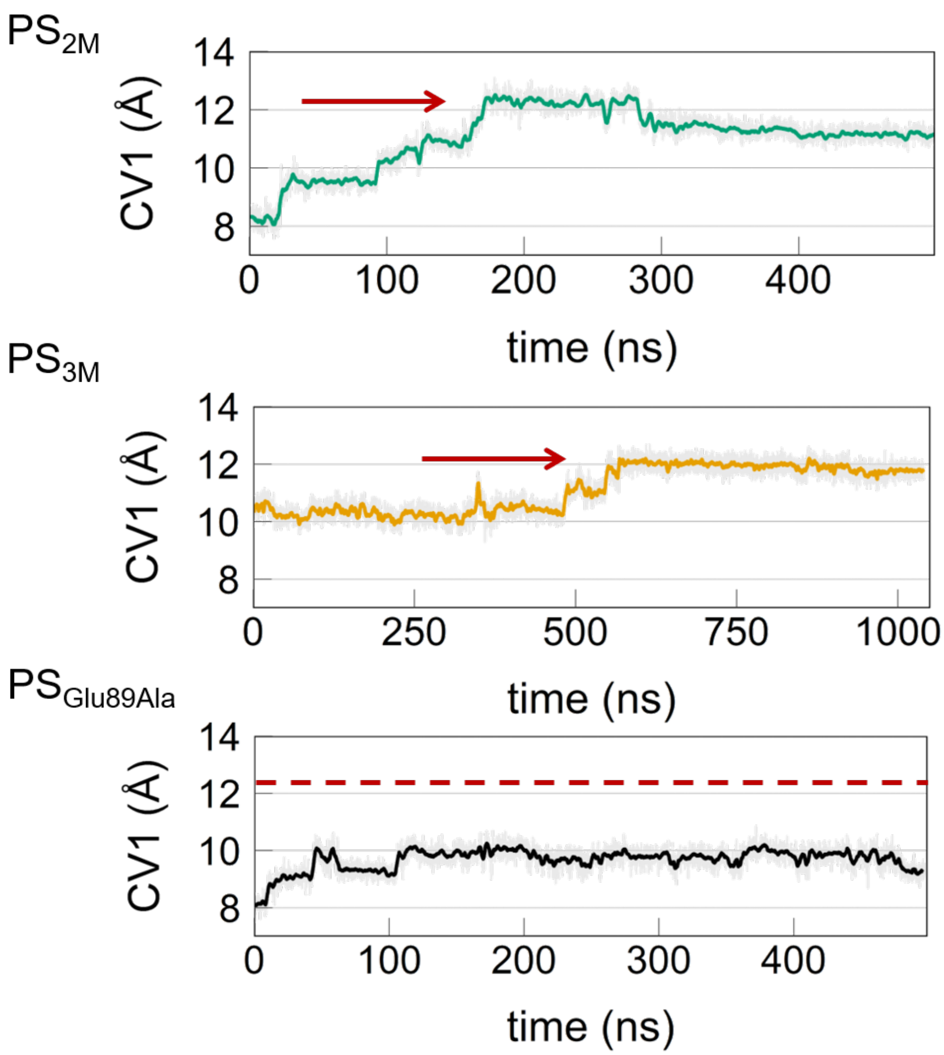

Figure S8. CV1 measures the distance between the centre of mass (COM) of the heavy atoms of AMP and the COM of the Cα of the aspartates in the first coordination shell of the two catalytic metal ions, i.e. Asp152, Asp171 and Asp173. (A) Schematic representation of CV1, exemplified using a snapshot taken from PS_{3M} simulations. (B) Frequency distribution of CV1 for PS_{2M} (green), PS_{3M} (orange), $PS_{Glu89Ala}$ (black). (C) Time evolution of CV1 for the Product State systems, PS_{2M} (green), PS_{3M} (orange), $PS_{Glu89Ala}$ (black).

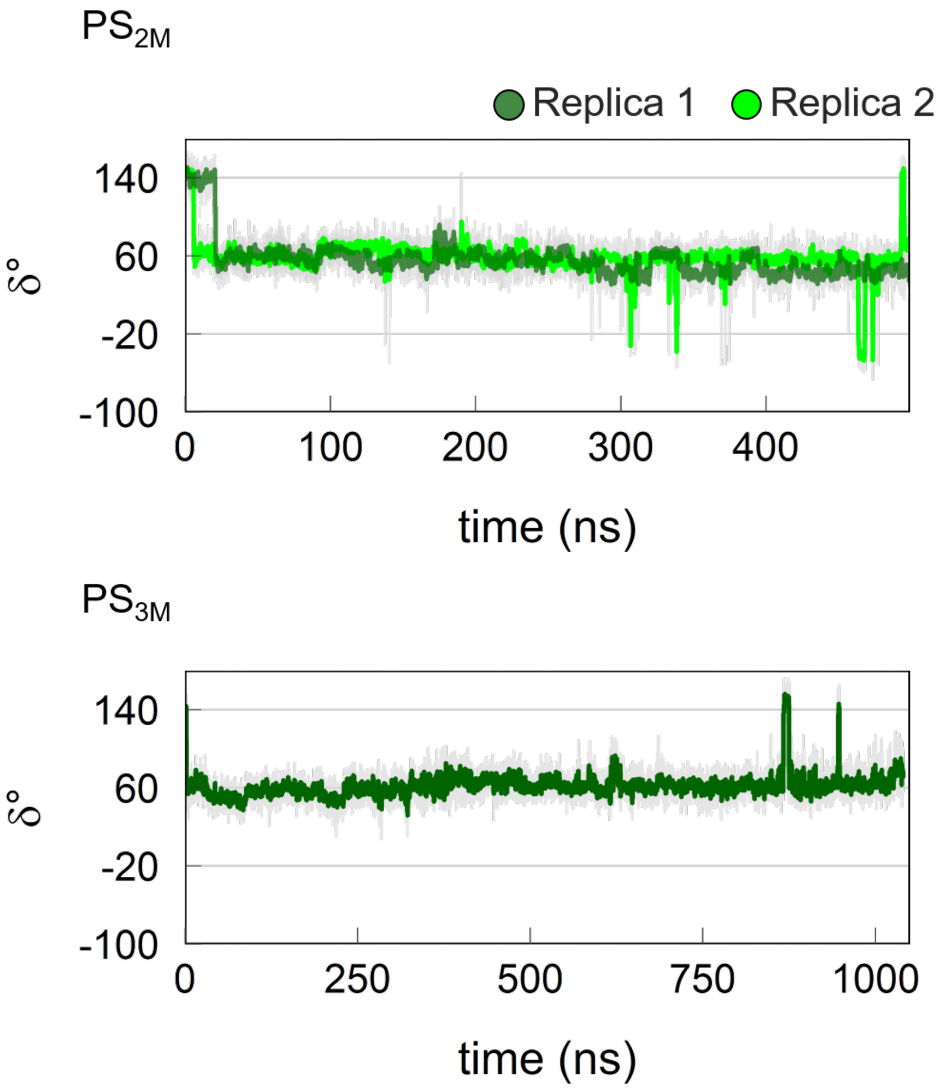

Figure S9. Time evolution of dihedral angle (δ), taken along C, Cα, Cβ, Cγ bonds, for the guide residue His36 for the PS_{2M} (top) and PS_{3M} (bottom) systems.

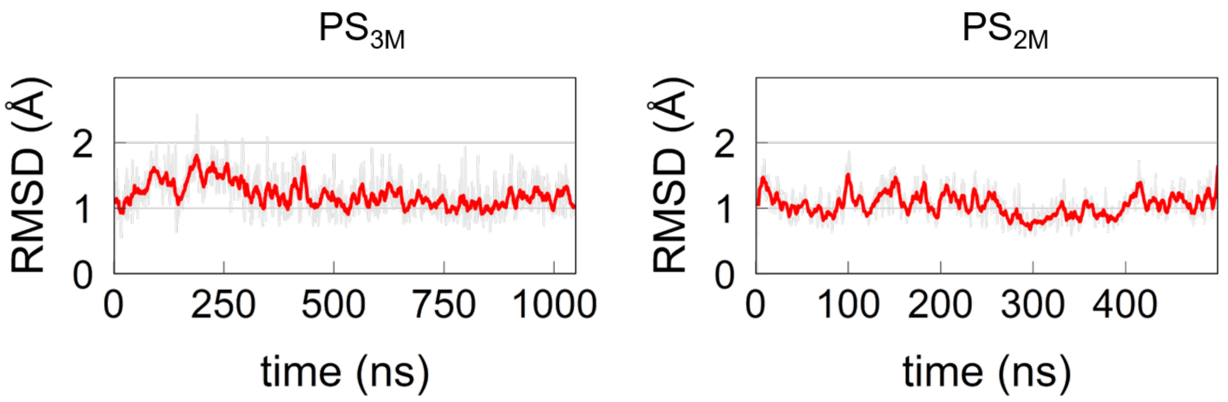

Figure S10. Time evolution of root mean square deviation (RMSD) of residues 80-125 (i.e. comprising the mobile arch), for the PS_{3M} (left) and PS_{2M} (right) systems.

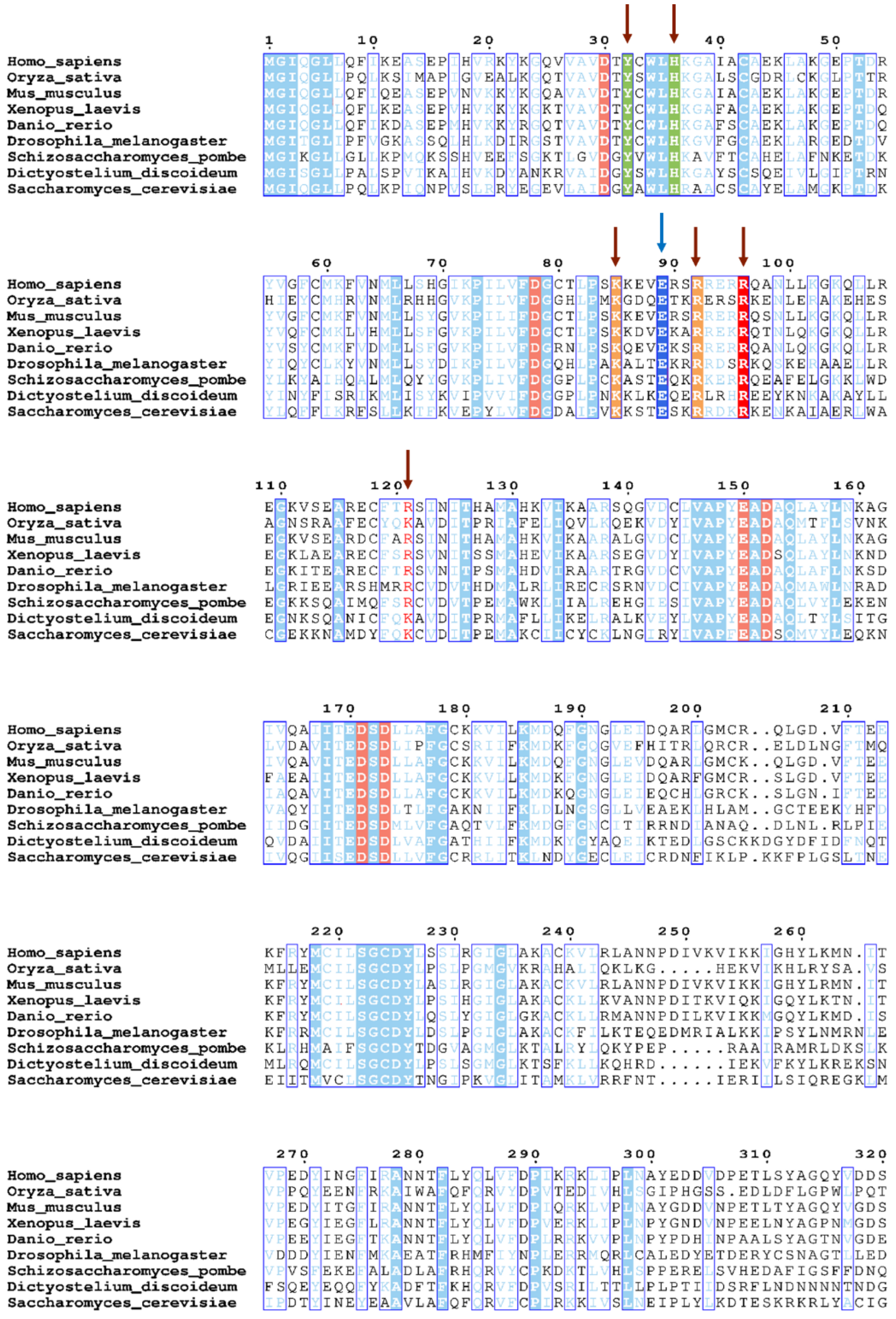

Figure S11. Sequence alignments of Exonuclease1 from 9 different organisms. Glu89 (blue) is strictly conserved across exonucleases as for the basic residues in the first and second coordination shell of MgA and MgB (light red), catalytic residues Lys85 and Arg92 (orange), guide residues, Tyr32 and His36 (green) and some of the proposed 'steering' residues Arg96 and Arg121. Conserved key residues are marked with arrows.

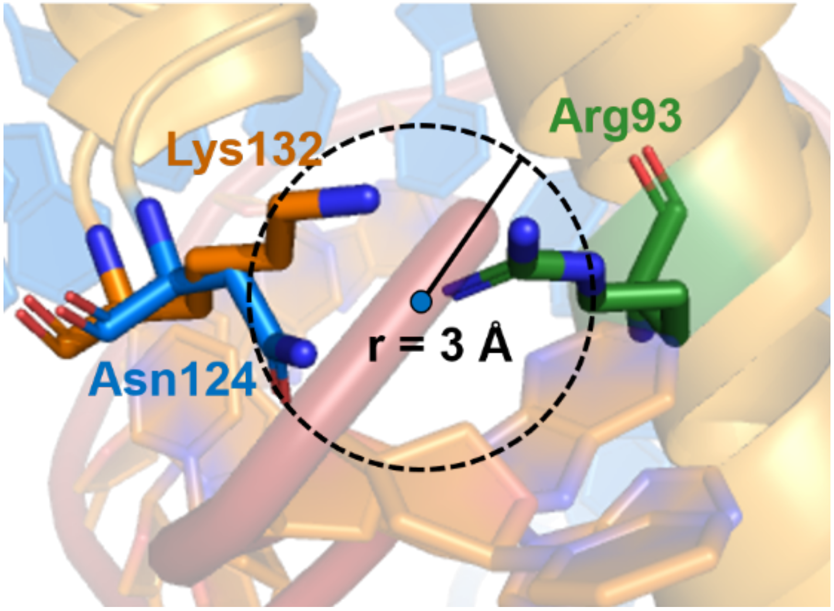

Figure S12. Structure alignment of the hExo1 crystal structure (PDB ID 5V0E) with the hFEN1 crystal structure (PDB ID 5KSE). The terminal guanidine, amide and amine groups of Arg93, Asn124 (in hExo1) and Lys132 (in hFEN1) residues are within a sphere of ~3 Å.

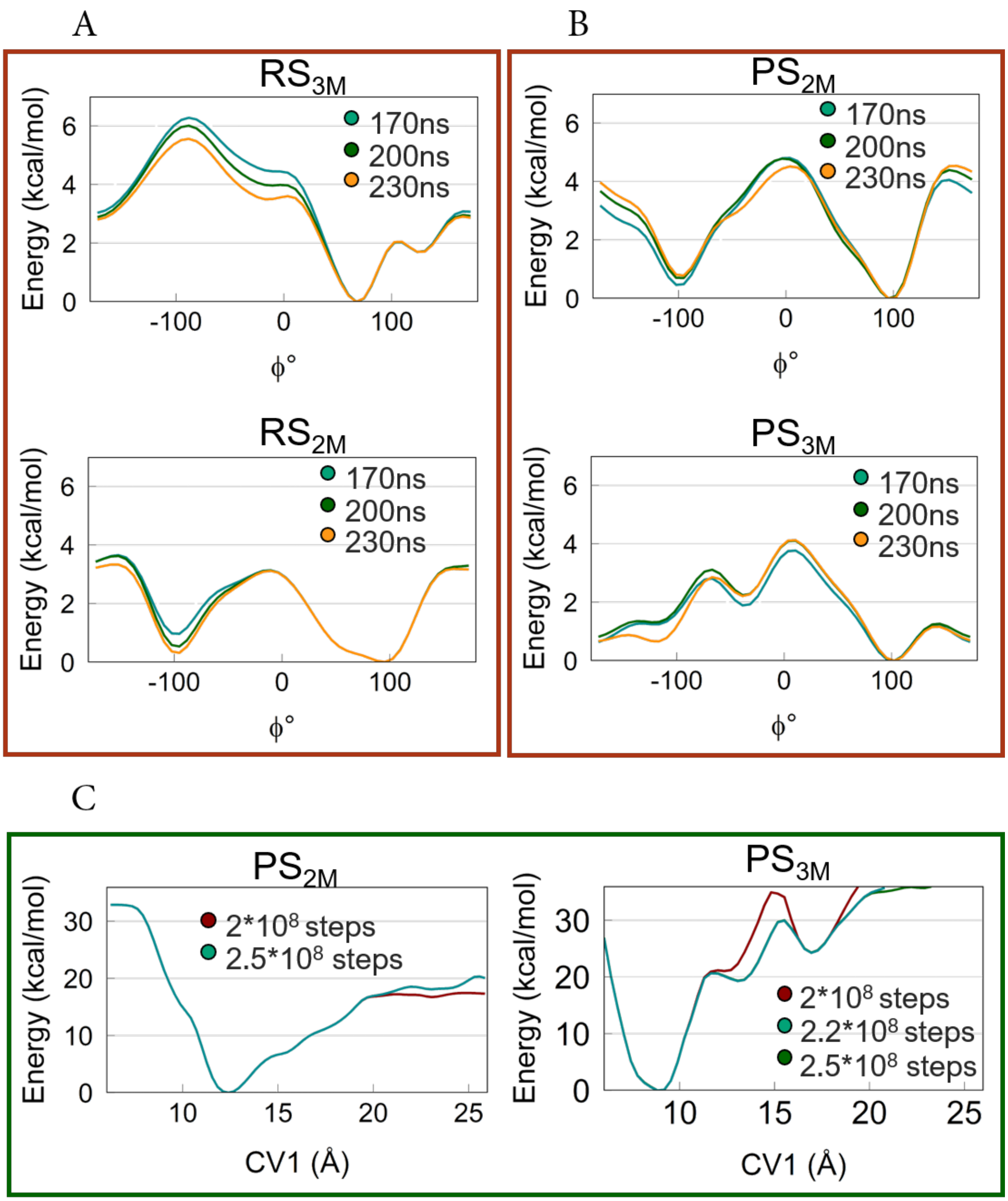

Figure S13. Convergence of the well-tempered metadynamics simulations for the Glu89 inner/outer flipping in the Reactant States (A), and in the Product States (B). (C) Convergence of the confined well-tempered metadynamics simulations for the leaving group unbinding process. Convergence was checked considering the energy values as a function of time. From ~170 ns to ~230 ns, no significant changes in energy for Glu89 flipping were detected. From ~2*10^8 steps to ~2.5*10^8 steps, no significant changes in energy for the leaving group release were detected.

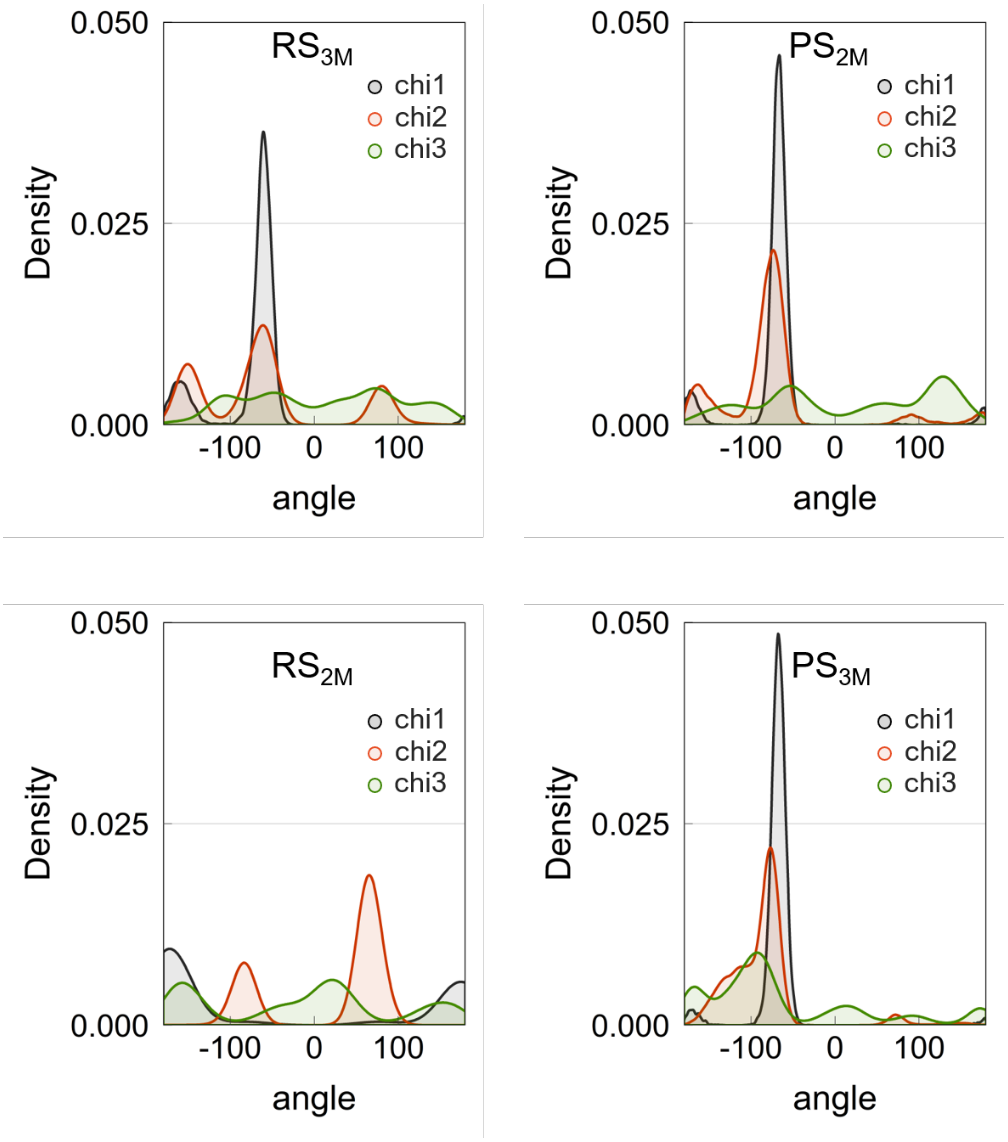

Figure S14. Population of rotamers (χ1, χ2, χ3) of Glu89 in RS_{3M}, RS_{2M}, PS_{3M} and PS_{2M}.

Movie S15. This movie shows the catalytic domain of hExo1 with colours for different motifs and the overall catalytic process of hExo1. The movie is composed by multiple trajectories extracted from force-field based MD simulations representing the recruitment mechanism of the third ion from the bulk in the Reactant State, and trajectories from metadynamics simulations for the departure of the leaving group from the catalytic site in the Product State. Hydrolytic incision step was inferred from crystal structures (i.e. PDB ID 5V06, 5V0A).

Table S16. The table summarizes all the simulations performed. Multiple replicas per system were run, as specified in the results section of the manuscript.

System name	**Method**	**Catalytic state**	***MgC**	**Mutation**
RS_{3M}	Force-field MD	Reactant state	Present	NA
RS_{2M}	Force-field MD	Reactant state	Absent	NA
PS_{2M}	Force-field MD	Product state	Absent	NA
PS_{3M}	Force-field MD	Product state	Present	NA
$RS_{Glu89Ala}$	Force-field MD	Reactant state	Absent	Glu89Ala
$PS_{Glu89Ala}$	Force-field MD	Product state	Absent	Glu89Ala
RS_{3M}	Well-tempered Metadynamic	Reactant state	Present	NA
RS_{2M}	Well-tempered Metadynamic	Reactant state	Absent	NA
PS_{2M}	Well-tempered Metadynamic	Product state	Absent	NA
PS_{3M}	Well-tempered Metadynamic	Product state	Present	NA
PS_{3M}	Confined Metadynamic	Product state	Present	NA
PS_{2M}	Confined Metadynamic	Product state	Absent	NA

*Present/Absent is referring to the starting point, when the minimization step starts.

REFERENCES

(1) Shi, Y.; Hellinga, H. W.; Beese, L. S. Interplay of Catalysis, Fidelity, Threading, and Processivity in the Exo- and Endonucleolytic Reactions of Human Exonuclease I. *Proc. Natl. Acad. Sci.* **2017**, *114* (23), 6010–6015.

(2) Case, D. A.; Betz, R. M.; Cerutti, D. S.; Cheatham, T. E.; Darden, T. A.; Duke, R. E.; Giese, T. J.; Gohlke, H.; Goetz, A. W.; Homeyer, N.; Izadi, S.; Janowski, P.; Kaus, J.; Kovalenko, A.; Lee, T. S.; LeGrand, S.; Li, P.; Lin, C.; Luchko, T.; Luo, R.; Madej, B.; Mermelstein, D.; Merz, K. M.; Monard, G.; Nguyen, H.; Nguyen, H. T.; Omelyan, I.; Onufriev, A.; Roe, D. R.; Roitberg, A.; Sagui, C.; Simmerling, C. L.; Botello-Smith, W. M.; Swails, J.; Walker, R. C.; Wang, J.; Wolf, R. M.; Wu, X.; Xiao, L.; Kollman, P. A AMBER 2017; University of California: San Francisco, **2017**.

(3) Piana, S.; Lindorff-Larsen, K.; Dirks, R. M.; Salmon, J. K.; Dror, R. O.; Shaw, D. E. Evaluating the Effects of Cutoffs and Treatment of Long-Range Electrostatics in Protein Folding Simulations. *PLoS One* **2012**, *7* (6), e39918.

(4) Parrinello, M.; Rahman, A. Polymorphic Transitions in Single Crystals: A New Molecular Dynamics Method. *J. Appl. Phys.* **1981**, *52* (12), 7182–7190.

(5) Bussi, G.; Donadio, D.; Parrinello, M. Canonical Sampling through Velocity Rescaling. *J. Chem. Phys.* **2007**, *126* (1), 014101.

(6) La Sala, G.; Riccardi, L.; Gaspari, R.; Cavalli, A.; Hantschel, O.; De Vivo, M. HRD Motif as the Central Hub of the Signaling Network for Activation Loop Autophosphorylation in Abl Kinase. *J. Chem. Theory Comput.* **2016**, *12* (11), 5563–5574.

(7) Laidler, K. J.; King, M. C. The Development of Transition-State Theory. *J. Phys. Chem.* **1983**, *87* (15), 2657–2664.

(8) Lee, B. I.; Wilson, D. M. The RAD2 Domain of Human Exonuclease 1 Exhibits 5′ to 3′ Exonuclease and Flap Structure-Specific Endonuclease Activities. *J. Biol. Chem.* **1999**, *274* (53), 37763–37769.
